# Topological and system-level protein interaction network (PIN) analyses to deduce molecular mechanism of curcumin

**DOI:** 10.1038/s41598-020-69011-0

**Published:** 2020-07-21

**Authors:** Anupam Dhasmana, Swati Uniyal, Anukriti, Vivek Kumar Kashyap, Pallavi Somvanshi, Meenu Gupta, Uma Bhardwaj, Meena Jaggi, Murali M. Yallapu, Shafiul Haque, Subhash C. Chauhan

**Affiliations:** 1 Department of Immunology and Microbiology, School of Medicine, University of Texas Rio Grande Valley, Edinburg, TX USA; 2 Department of Biosciences and Cancer Research Institute, Himalayan Institute of Medical Sciences, Swami Rama Himalayan University, Dehradun, India; 3 School of Biotechnology, Gautam Buddha University, Greater Noida, India; 4 Department of Biotechnology, TERI School of Advanced Studies, 10, Institutional Area, Vasant Kunj,, New Delhi, India; 5 Research and Scientific Studies Unit, College of Nursing and Allied Health Sciences, Jazan University, Jazan, Saudi Arabia

**Keywords:** Cancer, Cancer prevention

## Abstract

Curcumin is an important bioactive component of turmeric and also one of the important natural products, which has been investigated extensively. The precise mode of action of curcumin and its impact on system level protein networks are still not well studied. To identify the curcumin governed regulatory action on protein interaction network (PIN), an interectome was created based on 788 key proteins, extracted from PubMed literatures, and constructed by using STRING and Cytoscape programs. The PIN rewired by curcumin was a scale-free, extremely linked biological system. MCODE plug-in was used for sub-modulization analysis, wherein we identified 25 modules; ClueGo plug-in was used for the pathway’s enrichment analysis, wherein 37 enriched signalling pathways were obtained. Most of them were associated with human diseases groups, particularly carcinogenesis, inflammation, and infectious diseases. Finally, the analysis of topological characteristic like bottleneck, degree, GO term/pathways analysis, bio-kinetics simulation, molecular docking, and dynamics studies were performed for the selection of key regulatory proteins of curcumin-rewired PIN. The current findings deduce a precise molecular mechanism that curcumin might exert in the system. This comprehensive in-silico study will help to understand how curcumin induces its anti-cancerous, anti-inflammatory, and anti-microbial effects in the human body.

## Introduction

Naturally derived compounds possess enormous potential for medicinal and therapeutic actions for the management of diseases and curcumin is one of the most suitable examples among them. Curcumin is a yellowish-orange polyphenolic compound of turmeric/haldi (*Curcuma longa*), a herb often found in curry powder. The reason for the selection of curcumin for this study is, it is one of the important and thoroughly investigated naturally occurring bioactive compound, but its precise mode of action is still unclear. The global curcumin market was valued at $58.4 million in 2019 and is projected to reach $104.19 million by 2025. (https://www.alliedmarketresearch.com/press-release/curcumin-market.html & https://www.grandviewresearch.com/industry-analysis/turmeric-extract-curcumin-market). Earlier studies clearly exhibit the highly pleiotropic actions of curcumin as anti-cancerous^1,2^, anti-inflammatory^2,3^, anti-microbial^4^ , anti-oxidant^5^, cardio-protective^6^, radio-protective^7^ and many more that makes it an ideal ingredient in different medical and food applications. Curcumin’s market is expected to witness a huge development due to growth in consumer awareness regarding its therapeutic properties, and that’s why an intense scientific study are urgently needed to explore the precise mechanistic action of curcumin. Many research studies have been published in the past dealing with the mode of action of curcumin yet inconclusive, and still the puzzle remains unsolved. This pleiotropic potential of curcumin can be endorsed to its capability to interact with a huge interactome of biomolecular targets of cellular system that participate in multiple signalling pathways^8^. Thus, it is of huge interest to advert the key regulatory targets of curcumin in various patho-biological conditions.

Hartwell et al. (1999), have published an influential article entitled "From molecular to modular cell biology" on the new challenges of modern biology and pointed out an issue on the importance of constructing a general framework in which biological networks could be understood as a part of a complex modular organization of biomolecules of cellular system^9^. In modern molecular biology, antireductionism or zoom out approach should be followed by biologist, it should not be only focused on functioning of individual biomolecular components, this kind of approach leads our vision towards the reductionism or zoom in approach. The anti-reductionism view provides a bigger picture of biomolecular components system and how these components are interconnected through a complex web system of interactions, directing to a function of a living cell. To solve this kind of complex proteome/genome networking, researchers generally use system biology and graph theory approaches and principles. Systems biology is an interdisciplinary field that has capacity to bridges the gap between in vitro and in vivo models by the data produced from *omics* and to reveal the functional perceptive of all biomolecules present in any biological model^44^. Whereas, the study of a complex network called graph theory, which is an area of a branch of discrete mathematics and simplify the complex biological problems^43^.

The whole proteome is involved in important physiological and signalling tasks in the cellular system, and every expression of the protein modulates the whole interactome. They rarely act alone and mostly form dynamic protein–protein interaction (PPI) networks to achieve multi-functionality and various cellular signalling pathways^10,11^. PPIs are fundamental action for many cellular pathways^12,13^.

Whereas Gene ontology (GO) offers a compilation of well-defined biological processes, molecular functions and cellular components of gene products. GO enrichment is well accepted statistical scheme utilized to spot mutual links between proteins and annotations to GO^14^. Keeping the potential of above stated in silico approaches in view, topological parameters, network modulation of big interactions and GO investigation might offer a competent technique to exemplify the biomolecular mechanism of curcumin.

The background of this article is based on the identification and elucidation of the key regulatory proteins among the interactome of curcumin that may play critical role in whole communication path/or process. Topological and system-level protein interaction network approaches were applied to identify the most significant proteins and to analyse the molecular pharmacology of curcumin. PubMed database was screened and extracted for the biomolecular targets sets of curcumin. The protein interaction networks (PINs) of curcumin were constructed by STRING followed by Cytoscape programs, different network properties and module were analysed based on graph theory parameters like degree, clustering co-efficient, betweeness centrality distribution and most important bottle neck. The modulation was done using MCODE and seed proteins were identified, and further pathway enrichment was performed using ClueGO. Molecular docking simulation was performed to find the best binding affinity of curcumin with the most probable and key regulatory biomolecular targets of curcumin. At last, bio-kinetic and molecular dynamic analysis were executed to find the kinetic behaviour of the key regulatory proteins in the absence and presence of curcumin followed by molecular dynamics to identify the best and most stable binding confirmation of the complex (key regulatory protein + curcumin). Overall, the findings of this study will aid in providing a more clear and comprehensive reference for therapeutic application of curcumin in a systemic model.

## Results

### Construction of the network

**Figure 1:**
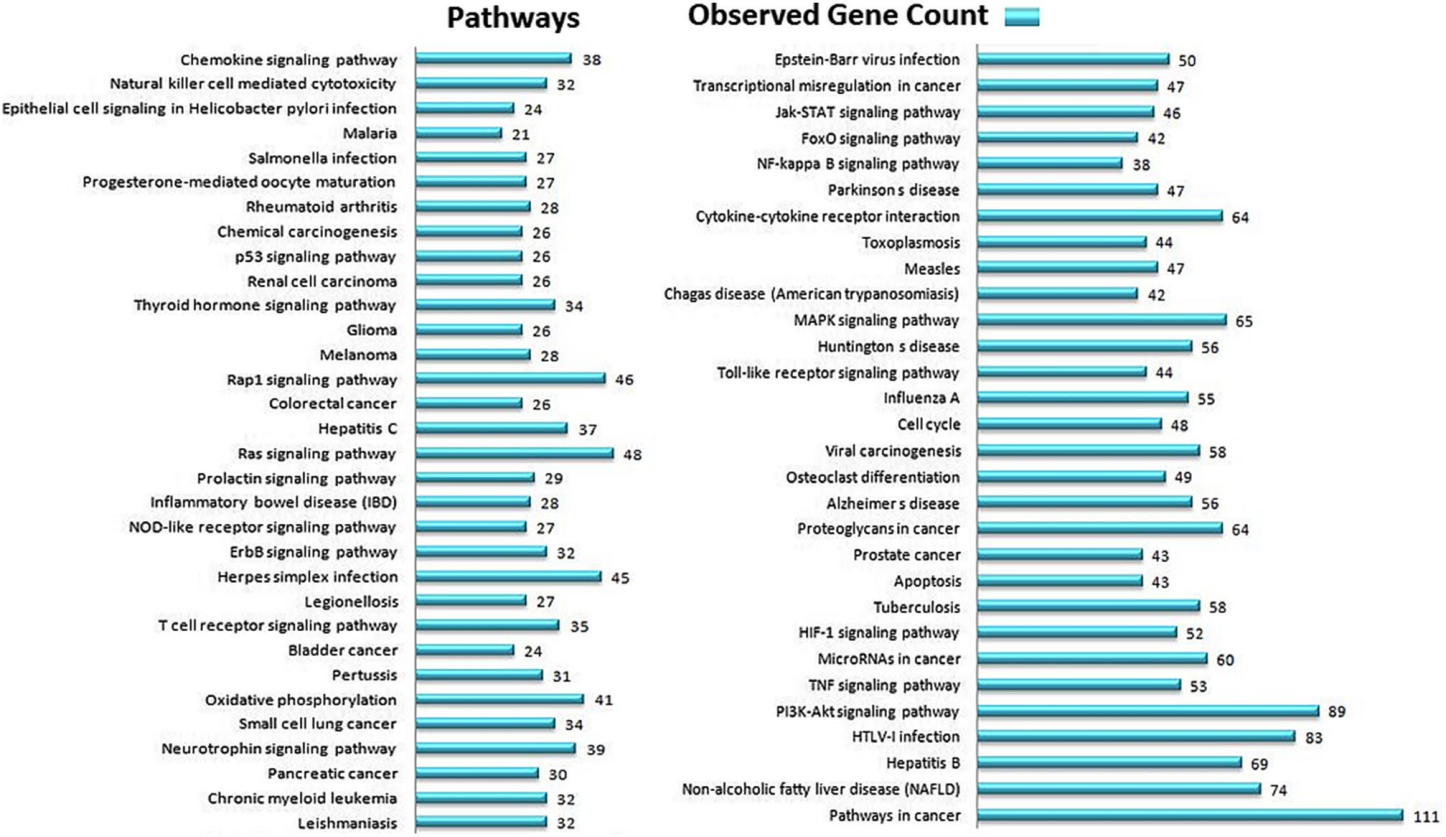
Classification of 62 enriched KEGG pathways for human diseases obtained during the PPI of curcumin associated proteins list of pathways was generated by STRING.

To provide an extensive insight view in the mechanistic action of curcumin in biological system, the network-based exploration was accomplished as shown in Supplementary Fig. 1. PESCADOR text mining PubMed web server with the key words "*Curcumin, Angiogenesis, Antioxidant, Apoptosis, Cancer, Cell Cycle, Cell Division, Free Radicals, Immunity, Inflammation, Inflammatory Response, Malignant, Migration, Neoplastic, Tumor, Wound healing*” were used to construct the abstract library that appraised the upshot of curcumin on gene/protein expression. A total of 2,228 reports were found from the above repository, and a total of 788 curcumin-altered genes/proteins were derived. STRING database was employed for generating the protein interaction network rewired by curcumin with 888 nodes (788 seed proteins & 100 connector proteins) (Supplementary Fig. 2). Those 888 proteins covered 30 different molecular functions (Supplementary Fig. 3), 62 different pathways (Fig. 1), 6,918 (physical or functional interactions) with an average node degree 1.56, average local clustering coefficient 0.479 and PPI enrichment p value < 1.0e^−16^.

### Topological features of interactome

**Figure 2:**
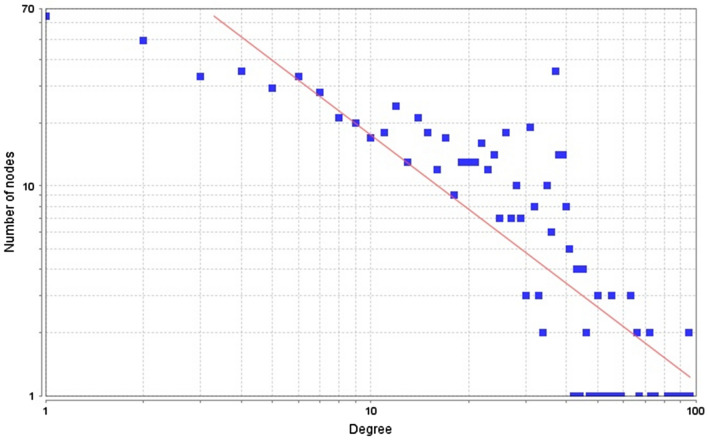
Node degree distribution. A power law of the form y = 258.52x − ^1.171^ was fitted (R-squared = 0.715) Graph generated by Cytoscape plugin Network Analyzer.

The PIN of any complex cellular system can be accessed from its topology and mutual connections. The topology can precisely expose the connectivity and interactions of biomolecules involved in diverse cellular and metabolic pathways^37,45^. In the present study, the protein/gene communication pathways were modified by curcumin, and Cytoscape and Network analyser plugin were used for topological analyses. Following the analysis by network analyser of Cytoscape, the number of links with each biomolecule called its ‘node degree’ was analysed. Node degree distribution is generally applied for differentiating the random and scale-free network topologies, whereas biological/cellular systems are generally scale-free^19,50^. In this study the degree exponent was calculated as 1.171 through fitting the node degree distribution curve (Fig. 2). If the number of a degree exponent is consistent with less than two, then it clearly signifies as a known biological network(s)^45^.

**Figure 3:**
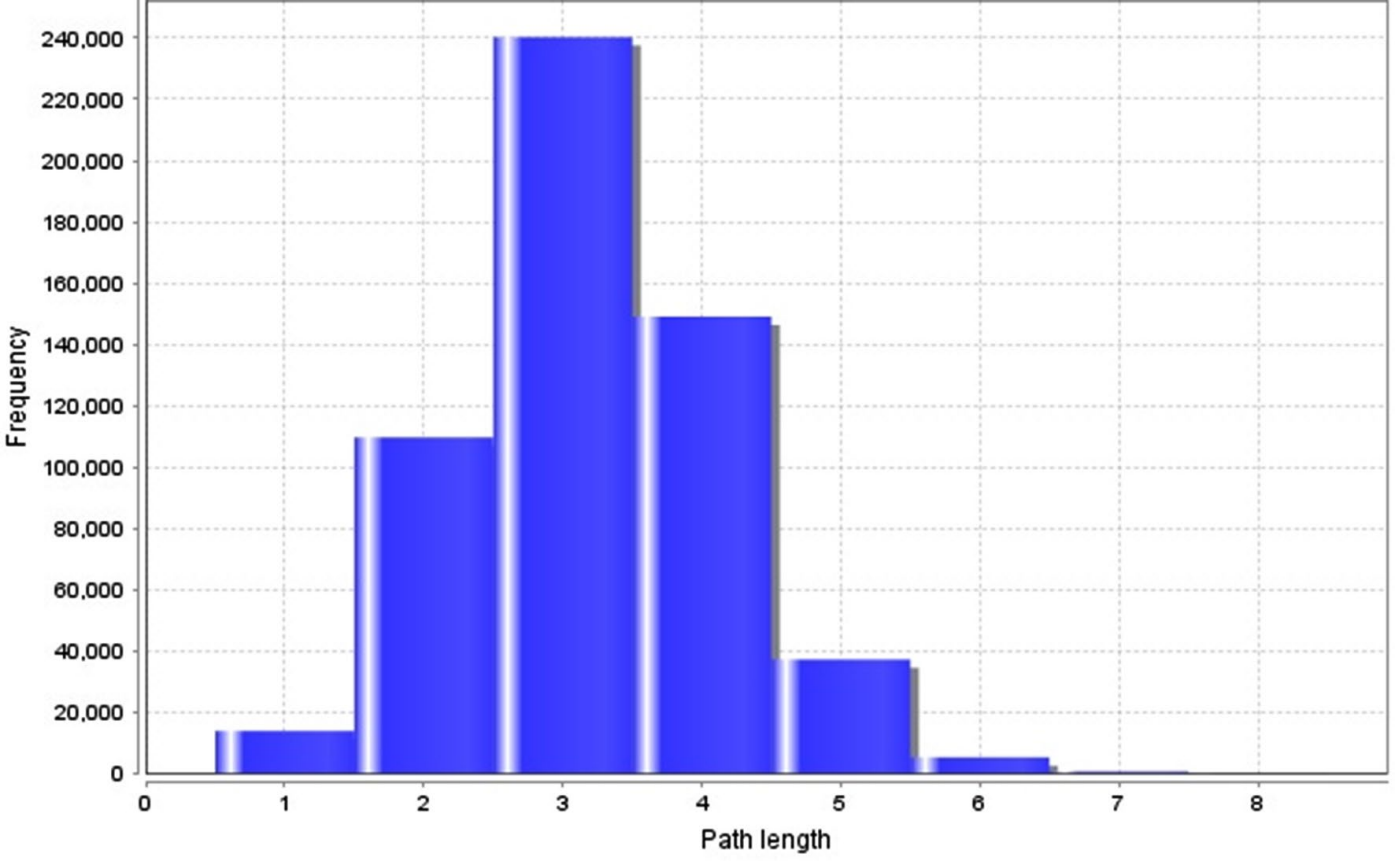
Characteristics Path length distribution is 3.188. Graph generated by Cytoscape plugin Network Analyzer.

**Figure 4:**
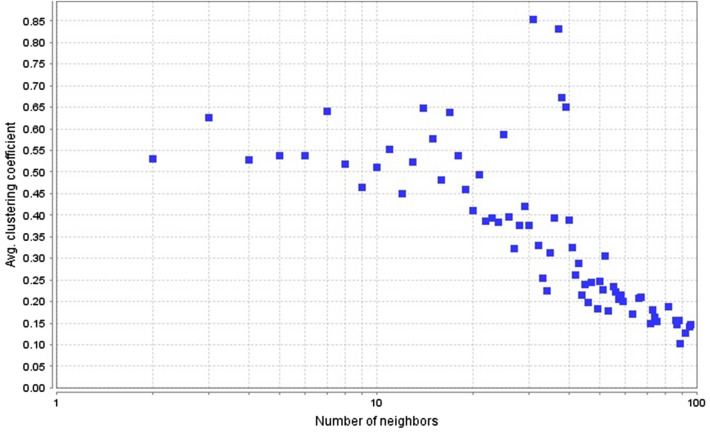
Clustering coefficient distribution is 0.472. Graph generated by Cytoscape plugin Network Analyzer.

Whereas the shortest path length for a PIN depicts the quantity of edges along the shortest paths among two nodes. On other side, closeness centrality is the inverse of the average shortest path (Fig. 3). These constraints specify the information transport competence and the overall navigability of the PIN^39,45^. The characteristic path length for curcumin-rewired PIN was 3.188. Clustering coefficient represents the closeness of nodes and neighbours and the hierarchical modularity of the PIN, and is used to spot the possible functional modules and uncover the molecular complexes or signalling pathways in the PIN^19,22,45^. For the curcumin-rewired network, the clustering coefficient distribution was determined as 0.472 (Fig. 4).

### Analysis of sub-clustering and gene ontology enrichment

MCODE plugin was used to recognize 25 modules from the PIN rewired by curcumin, (Supplementary Fig. 4). Red and yellow nodes indicated seed nodes and connector proteins, respectively.

Modularization was performed to remove the noise (yellow connector proteins from the whole curcumin interactome), screening of a huge data set for selecting the most important seed proteins in interactome and enrich the biomolecular pathways of the selected seed proteins. By using MCODE plugin in Cytoscape, the total curcumin-rewired network was divided into 25 modules. ClueGO plugin was used to determine the biological function of each module. A total of 37 pathways distributed in 25 modules and 195 seed proteins were recognized (Supplementary Table 1a and b gives the description of all 195 seed proteins with their impacts in the presence of curcumin).

### PIN construction, topological and GO analysis of final selected seed proteins

**Figure 5:**
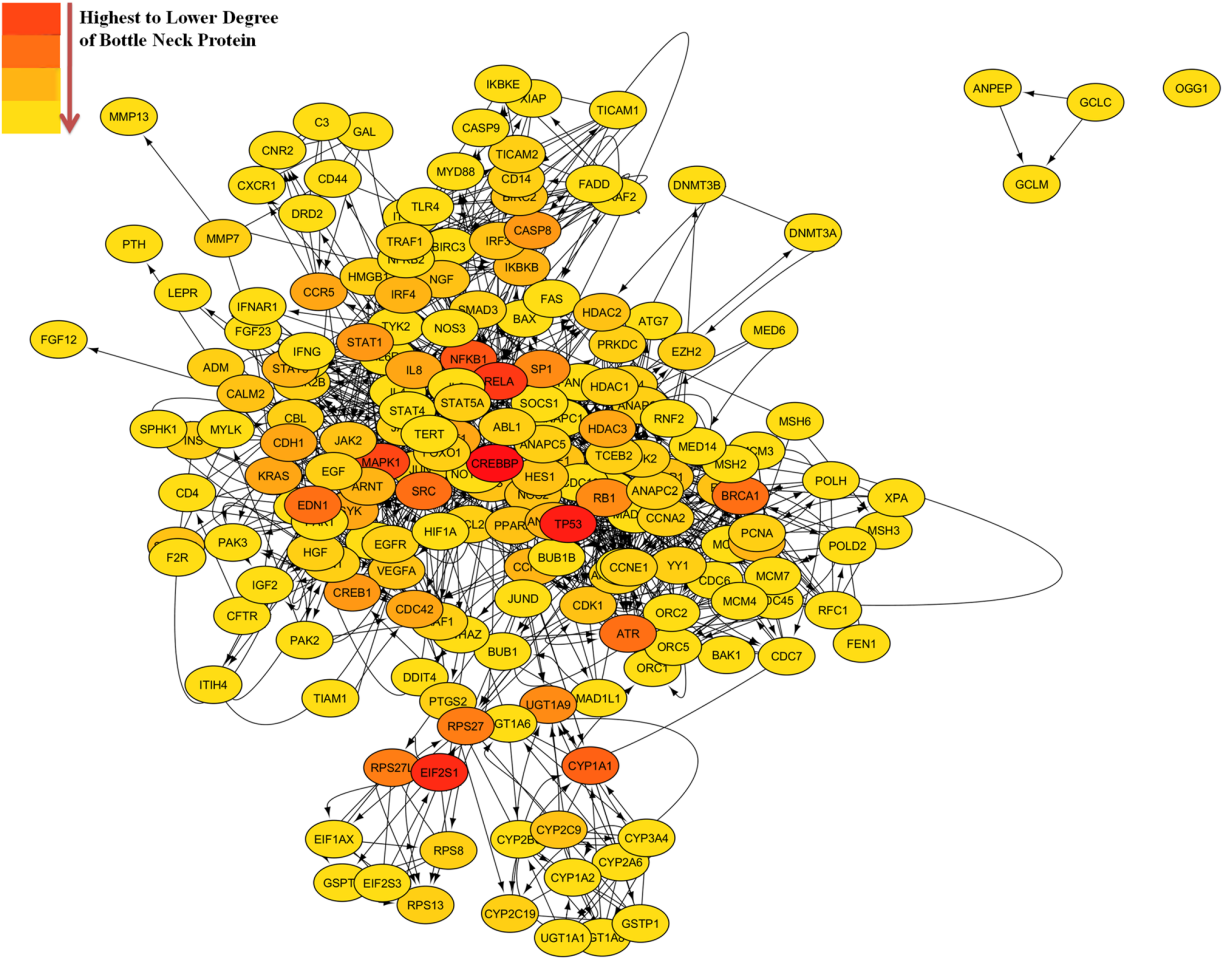
Final selected PIN of modulated seed proteins and connector proteins (Figure generated from Cytoscape) obtained from STRING**.**

Finally selected 195 curcumin-altered seed proteins were evaluated again by using STRING database and these seed proteins were integrated into the total PIN rewired by curcumin with 295 nodes (195 seed proteins & 100 connector proteins) (Fig. 5). The PIN generated string network setting was ‘evidence based’, extracting furthermore information through ‘experiments and curated databased’. We have selected the ‘high confidence score option of 0.9′ and have selected ‘50–50 interactors option in 1st and 2nd shells’. Following are the statistical details of curcumin rewired PIN: the number of nodes were 295 (195 seed proteins and 100 connectors), average node degree was 17.7, average clustering coefficient was 0.641 and PPI enrichment p-value was < 1.0e−16. Cytohubba plugin of Cytoscape was used for the topological analysis like bottle neck, betweenness, closeness, clustering coefficient and degree. By using the above said parameters top 10 bottle neck scored, 22 (21 seed proteins and 1 connector protein 'RB1’) proteins were selected (their clustering coefficient was less than 0.5) as shown in Table 1.

**Table 1:** 10 best bottle neck scores of seed and connector proteins. The selection was done on the basis of higher bottle neck score and clustering coefficient (less than 0.5, these proteins were considered as a date hub proteins).

S. No	Name	Bottle neck	Betweenness	Closeness	Clustering coefficient	Degree
1	CREBBP	52	13,223.35	155.8333	0.15837	50
2	TP53	22	7,640.317	148.4167	0.17639	38
3	RELA	14	4,458.032	149.0833	0.20863	51
4	MAPK1	13	4,779.75	146.5	0.18316	47
5	NFKB1	11	3,211.738	144.8333	0.22304	44
6	CYP1A1	10	4,831.488	110.8667	0.43137	18
7	SRC	9	2,899.138	135.6667	0.17424	33
	BRCA1	9	1537.711	128.1	0.38413	36
	EDN1	9	684.0157	123.8333	0.29412	18
	ATR	9	647.5952	124.0667	0.38391	30
8	RB1	7	2,625.42	142.8333	0.29445	38
	SP1	7	391.5314	130.6667	0.34762	21
9	CASP8	6	2,299.261	123.3667	0.4152	19
	STAT1	6	1,012.291	130.1667	0.36594	24
	CREB1	6	597.4255	121.6667	0.30882	17
10	AKT1	5	2,328.794	137.4167	0.17094	27
	IL8	5	2,227.319	121.1667	0.4058	24
	CDH1	5	1,162.138	113	0.34848	12
	KRAS	5	917.6512	120.8333	0.25692	23
	SYK	5	796.2624	123.6667	0.24265	17
	CCR5	5	669.6932	108.3667	0.42222	10
	HDAC3	5	471.1578	124.3333	0.35263	20

**Figure 6:**
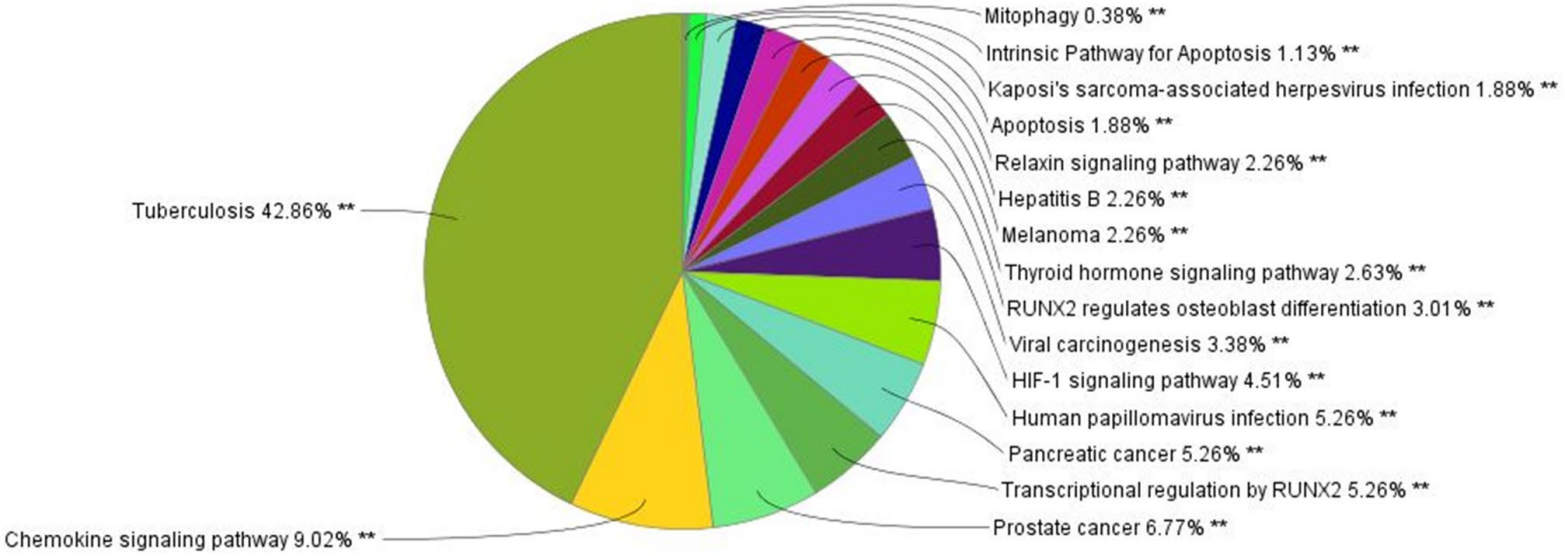
ClueGO results of GO functional enrichment of key proteins.

Finally, the key 22 proteins underwent for GO functional annotation and enrichment analysis. Gene Ontology (GO) enrichment analysis was performed by the ClueGO plugin (version 2.5.2) according to KEGG and Reactome database **(**Fig. 6 and Table 2). From this ClueGO plugin study, 17 GO groups were generated (from 0 to 16), with their GO ID, GO term and associated genes.

**Table 2:** GO functional group analysis with their associated genes (data generated by ClueGO plugin).

GOID	GO term	GO groups	Associated genes found
KEGG:04137	Mitophagy	Group00	[KRAS, RELA, SP1, SRC, TP53]
R-HSA:10906	Intrinsic pathway for apoptosis	Group01	[AKT1, CASP8, TP53]
KEGG:05167	Kaposi's sarcoma-associated herpes virus infection	Group02	[AKT1, CASP8, CCR5, CREB1, CREBBP, CXCL8, KRAS, MAPK1, NFKB1, RB1, RELA, SRC, STAT1, SYK, TP53]
KEGG:04210	Apoptosis	Group03	[AKT1, CASP8, KRAS, MAPK1, NFKB1, RELA, TP53]
KEGG:04926	Relaxin signalling pathway	Group04	[AKT1, CREB1, EDN1, KRAS, MAPK1, NFKB1, RELA, SRC]
KEGG:05161	Hepatitis B	Group05	[AKT1, CASP8, CREB1, CREBBP, CXCL8, KRAS, MAPK1, NFKB1, RB1, RELA, SRC, STAT1, TP53]
KEGG:05218	Melanoma	Group06	[AKT1, CDH1, KRAS, MAPK1, RB1, TP53]
KEGG:04919	Thyroid hormone signalling pathway	Group07	[AKT1, CREBBP, HDAC3, KRAS, MAPK1, SRC, STAT1, TP53]
R-HSA:8940973	RUNX2 regulates osteoblast differentiation	Group08	[HDAC3, MAPK1, RB1, SRC]
KEGG:05203	Viral carcinogenesis	Group09	[CASP8, CCR5, CREB1, CREBBP, HDAC3, KRAS, MAPK1, NFKB1, RB1, RELA, SRC, SYK, TP53]
KEGG:04066	HIF-1 signalling pathway	Group10	[AKT1, CREBBP, EDN1, MAPK1, NFKB1, RELA]
KEGG:05165	Human papilloma virus infection	Group11	[AKT1, ATR, CASP8, CREB1, CREBBP, HDAC3, KRAS, MAPK1, NFKB1, RB1, RELA, STAT1, TP53]
KEGG:05212	Pancreatic cancer	Group12	[AKT1, KRAS, MAPK1, NFKB1, RB1, RELA, STAT1, TP53]
R-HSA:8878166	Transcriptional regulation by RUNX2	Group13	[AKT1, HDAC3, MAPK1, RB1, SRC, STAT1]
KEGG:05215	Prostate cancer	Group14	[AKT1, CREB1, CREBBP, KRAS, MAPK1, NFKB1, RB1, RELA, TP53]
KEGG:04062	Chemokine signalling pathway	Group15	[AKT1, CCR5, CXCL8, KRAS, MAPK1, NFKB1, RELA, SRC, STAT1]
KEGG:05152	Tuberculosis	Group16	[AKT1, CASP8, CREB1, CREBBP, MAPK1, NFKB1, RELA, SRC, STAT1, SYK]

### Molecular docking of curcumin with key proteins

Auto Dock 4.0, was used for the molecular docking of final 12 possible key proteins, which were screened on the basis of each proteins participated in max no. of pathways. MAPK1/Erk2 showed the highest and strongest binding affinity with curcumin (− 8.43 kcal/Mol), followed by STAT1 (− 7.68 kcal/Mol), KRAS (− 7.48 kcal/Mol), P53 (− 6.57 kcal/Mol), CREBBP (− 6.29 kcal/Mol), RELA (− 6.07 kcal/Mol), AKT1 (− 6.04 kcal/Mol), CASP8 (− 5.81Kcal/Mol), CREB1 (− 5.73 kcal/Mol), NFKB1 (− 5.72 kcal/Mol), RB1 (− 5.34 kcal/Mol) and SRC (− 4.85 kcal/Mol) as shown in Supplementary Table 2.

### Molecular dynamics simulation analysis of MAPK1 and curcumin

**Figure 7:**
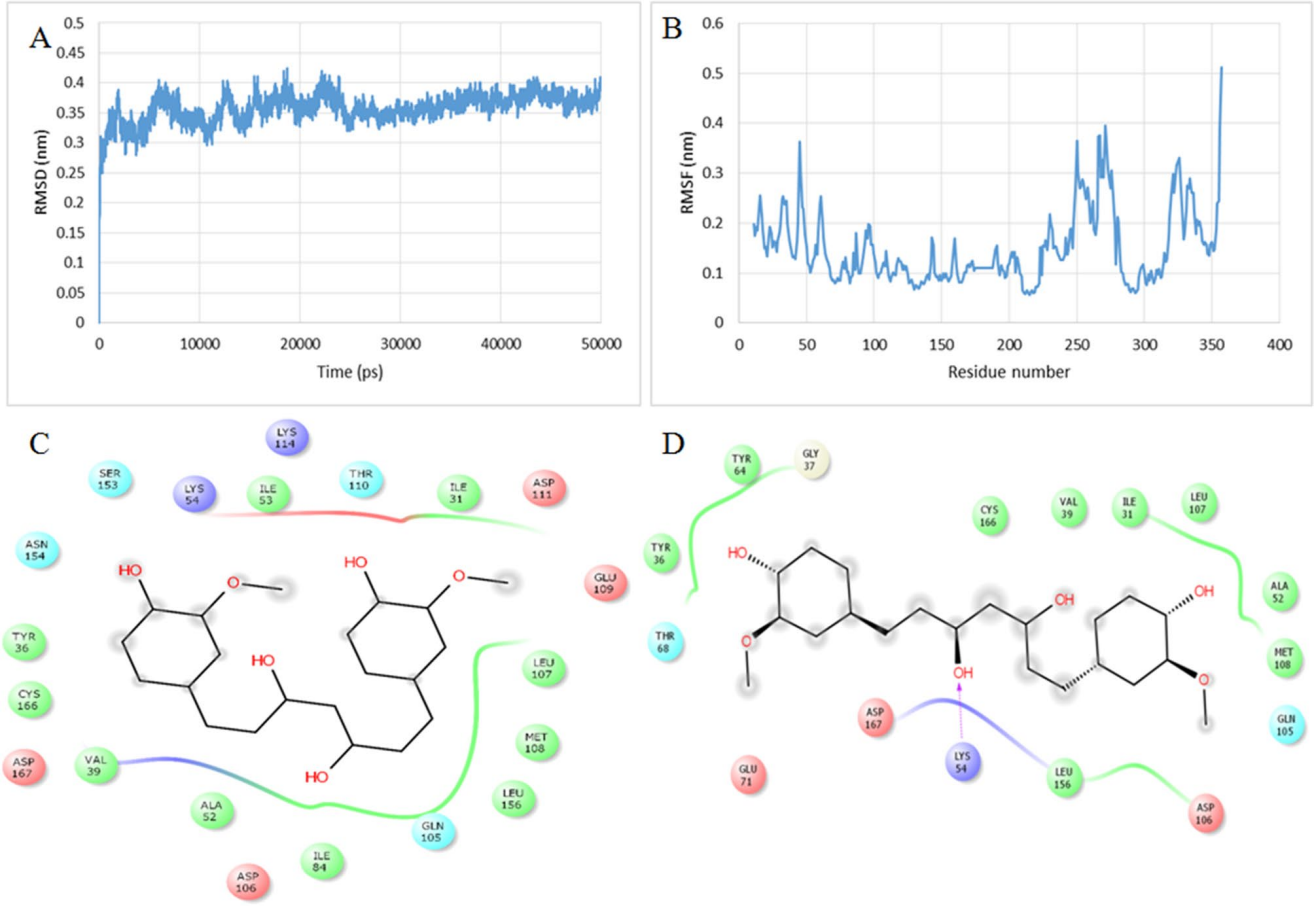
(**A**) Backbone RMSD of curcumin bound MAPK1 during 50000 ps molecular dynamics trajectory; (**B**) Per residue fluctuations of curcumin bound MAPK1 during 50000 ps molecular dynamics; (**C**) Pre-MD interactions of curcumin with MAPK1; (**D**) Post-MD interactions of curcumin with MAPK1.

Molecular dynamics simulations of the docked MPAK1-curcumin complexes were carried out to analyse the effect of curcumin on conformational dynamics of MAPK1 protein in curcumin bound form. The dynamic behaviour of curcumin bound MAPK1 complex during molecular dynamics simulation was analysed for the stability via RMSD calculations, which showed that the system reached to a perfect equilibrium at around 25,000 pico-second and remains stable till the end of the simulation with minimal fluctuations of 0.05 nm (Fig. 7A). Per residue amino acid flexibility analysis was done to find the effect of curcumin binding on MAPK1 flexibility^40^. Root mean square fluctuation (RMSF) of each amino acid residue was calculated with c-alpha atoms ^41^ and the RMSF curves showed that curcumin bound MAPK1 residues had lower RMSF values, which indicated the stable binding (Fig. 7B). Figure 7C and D represents hydrogen and hydrophobic interactions before and after the molecular dynamic simulations.

### Inhibitory analysis of curcumin on the bio-kinetic simulation of MAPK cascade

**Figure 8:**
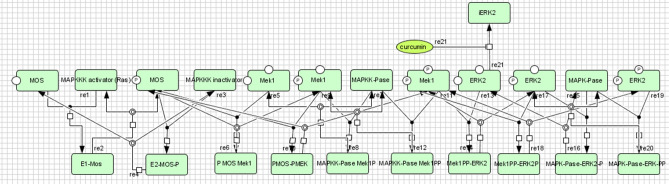
Demonstrate the MAPK cascade and the inhibition of MAPK1/Erk2 by curcumin.

**Figure 9:**
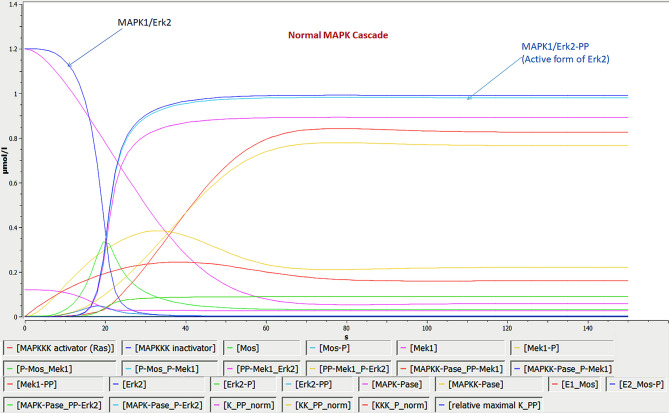
Bio-kinetic simulation of MAPK cascade in normal condition.

**Figure 10:**
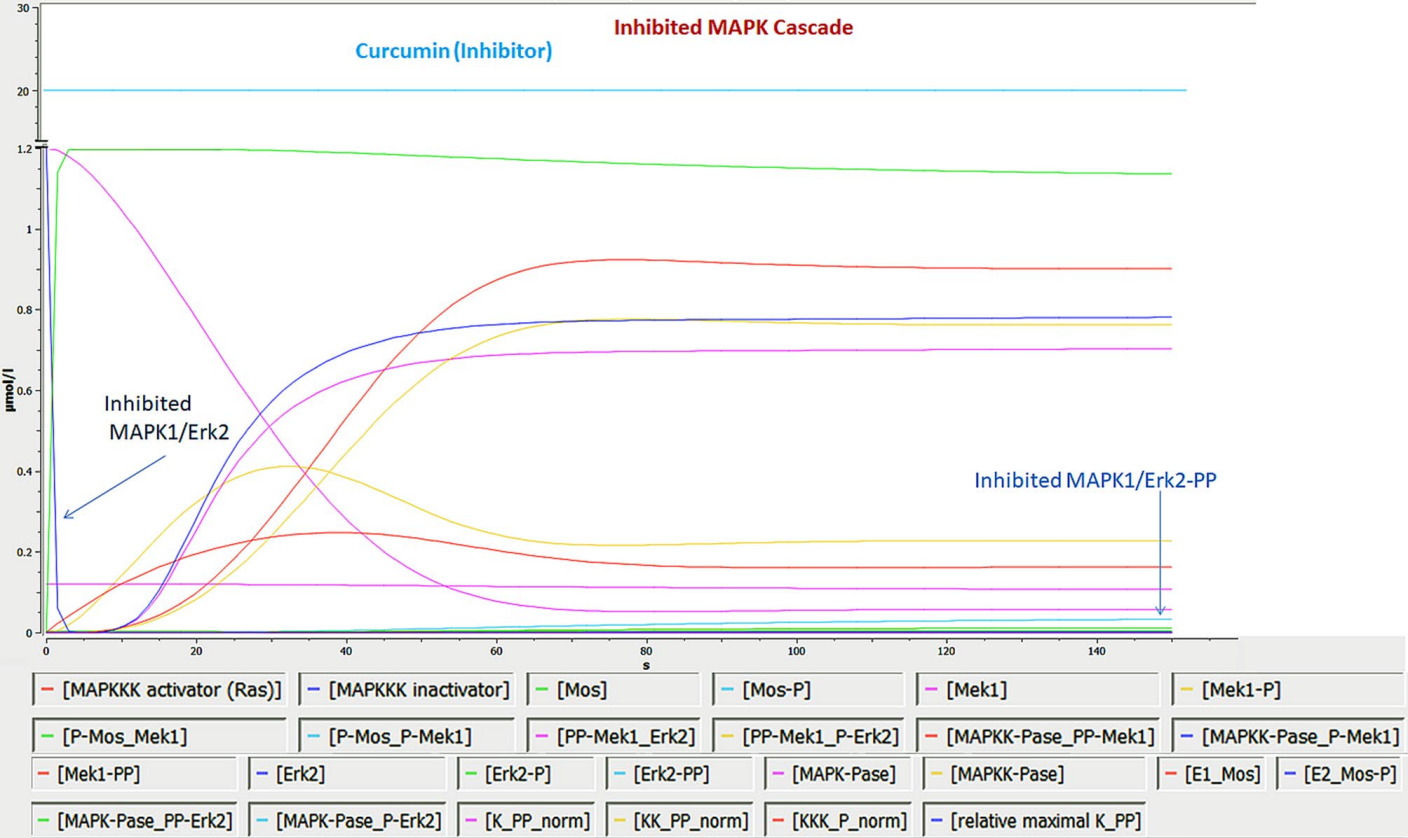
Bio-kinetic simulation of inhibitory impact of curcumin on MAPK cascade.

For the construction of interacting molecules of MAPK cascade and inhibitory impact of curcumin, Cell Designer was used as shown in Fig. 8. To carry out bio-kinetics study in the presence/absence of curcumin, each entity values were assigned in the form of concentration, which were mentioned in BioModel database (BIOMD0000000009); 20 µM concentration was assigned for curcumin^42^ and the whole kinetics was applied for the entire pathway using mass action kinetics equation. The current study predicted the role of entities involved in the activation MAPK cascade from its inactive MAPK1/Erk2 (conc. 1.2 µM/L in 0 s, 1.19995 µM/L in 1.5 s and 0.0010782 µM/L in 150 s) to active phosphorylated MAPK1/Erk2 form (0.981202 µM/L in 150 s) (Fig. 9) and changes in MAPK bio-kinetic, followed by the inhibition of MAPK1/Erk2 (conc. 1.2 µM/L in 0 s, 0.0602204 µM/L in 1.5 s and 0.000465626 µM/L in 150 s) and inhibited phosphorylated MAPK1/Erk2 (0.0331032 µM/L in 150 s) in the presence of curcumin (20 µM) using systems biology approach (Fig. 10). X-axis symbolizes the transition time and Y-axis concentration of the entities.

## Discussion

In the present study, with the help of text mining software PESCADOR, we have tried to extract out all the published genes/proteins that have been reported to show alterations on interaction with curcumin. Using graph theory^43^ and systems biology^44^ approaches, 888 (788 seed proteins and 100 connecter proteins) nodes containing global curcumin–rewired PIN was constructed by using STRING db software. Curcumin induces an intense and highly connected PIN, which clearly shows broad influence of curcumin on various biological systems. The network was further analysed for its topological properties based on the tools of graph theory. Network analyser, a plugin of Cytoscape software was used for the analysis. The most important topological property of any network is its node degree distribution that tells, whether a network is scale free or not. The topological property analysis of curcumin rewired network clearly depicted that it is a scale free network in which all the nodes were free to interact globally. The degree distribution of a scale-free network in a logarithmic scale, can observe how it fits with a power-law line, having a less number of biomolecules/nodes with high degree (the hubs) and a large number of biomolecules/nodes with a low degree. The node degree distribution of the curcumin-rewired PINs was in accord with the power law, indicating that the constructed network had scale-free topology. The significance of the hubs is indicated by the exponent of the power law, which distinguishes between biological and non-biological networks. Usually, a small exponent means significant central hubs^19,45^. In cellular systems, the hubs with an exponent of < 2 usually have important roles, which demonstrated that the number for degree exponent less than two is consistent with known biological networks^19,38,39,45^. The degree exponent of this network was 1.171, which clearly signified that the generated PIN was biological and scale-free.

Another important topological properties are shortest path length and average clustering coefficient, which came out to be 3.188 and 0.472. The path length shows that the information in the network travels at a fast pace and clustering coefficient depicts the tendency of nodes to form a cluster. From small path lengths and clustering coefficient we can infer that the network is packed with nodes that pass on the information quickly and small average clustering coefficient depicts that the network had high number of nodes, but holds lesser no. of connection, so there are chances of binding of drug or other ligands^47^.

Following the topological analysis of the network, modulation using MCODE^50^ and pathway enrichment using ClueGo^51^ was performed. On modularization 25 modules were obtained and on ClueGo analysis 37 GO pathways were identified from the global networks, which are mentioned in Supplementary Table 1. The majority of the pathways belong to the pathological conditions, particularly in, ageing, stress, metabolic pathways, cancer, DNA replication, apoptosis, inflammatory, infectious and allergic diseases etc., which suggests that these signalling pathways may be the main contributing factors to the pharmacological effects of curcumin. The role of modularization was to sort the noise or less important proteins from the global curcumin-rewired PIN, that led us towards the exploration of more precise key regulatory proteins, which regulate the interactome of curcumin. The modularization was executed for spotting the clusters of nodes that are strongly connected with each other but are less connected with the nodes outside. The modularization possibly characterize the node from the important complexes like signal transducer, and are the network core in terms of functionality^45^. After modularization of 195 seed proteins, STRING was used again to construct a core PIN of 295 nodes (195 seed proteins and 100 connector proteins). The motive of this PIN construction was to reconstruct and find other important connector proteins that might play an important role in exhibiting the pharmacological action of curcumin and were not reported earlier. In continuation of this approach, graph theory based topological analysis was performed and further protein sorting was done on the basis of top 10 bottle neck score and clustering coefficient of less than 0.5. These proteins were considered as date hub proteins. This sorting parameter led to the identification of 22 possible key proteins (21 seed proteins and 1 connector protein, RB1) among 295 proteins. These 22 possible key proteins (CREBBP, TP53, RELA, MAPK1, NFKB1, CYP1A1, SRC, BRCA1, EDN1, ATR, RB1, SP1, CASP8, STAT1, CREB1, AKT1, IL8, CDH1, KRAS, SYK, CCR5, HDAC3) referred as top 10 bottleneck scorer (from 52 to 5) proteins. The characteristics of bottlenecks proteins are with a high betweenness centrality i.e., PIN nodes with many “shortest paths” going through them, similar to bridges and tunnels on a highway map, this character considered the fast communication between the nodes. These are possibly very crucial proteins in the PIN and much more significant indicator of essentiality than degree (hub proteins)^46^. CREBBP, TP53, RELA, MAPK1 and NFkB1 were best top five possible key proteins with bottleneckness score of 52, 22, 14, 13 and 11, respectively.

Furthermore, another important topological feature of the selection of these possible key proteins is clustering co-efficient (Pearson correlation coefficient), which is less than 0.5, was assumed and classified the date hubs. These date proteins are important in PIN but holds less no. of connection, so the chances of the binding of a drug or any other ligand will be higher than the party proteins (party proteins: co-efficient more than 0.5 and show high degree of co-expression with interaction nodes/partner, are assumed to interact at the same time with their interaction nodes/partners)^47^.

**Figure 11:**
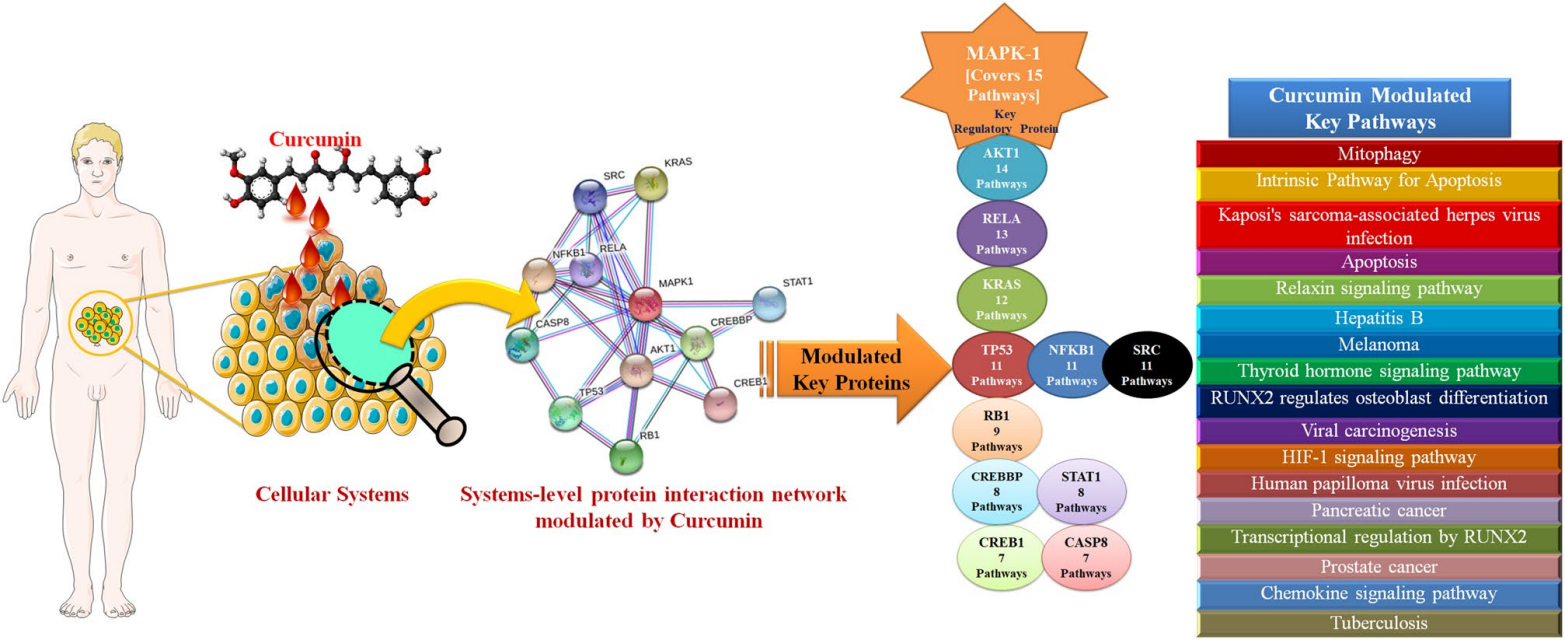
Graphical representation of curcumin's impact on systems level proteins interaction network with their respective key proteins, number of associated and enriched pathways.

Twenty-two (22) possible key proteins underwent to GO functional annotation and enrichment analysis. In this study, ClueGO plugin generated 17 GO groups (from 0 to 16), including GO ID, GO term/Pathways and associated genes/proteins as shown in Table 2. Among all the associated proteins, MAPK1 was covering and associated with highest 15 pathways as shown in Fig. 11, followed by AKT1; 14, RELA; 13, KRAS; 12, TP53; 11, NFKB1; 11, SRC; 10, RB1; 9, CREBBP; 8, STAT1; 8, CREB1; 7, CASP8; 7, HDAC3; 5, SYK; 3, CCR5; 3, EDN1; 2, SP1; 1, CDH1; 1, ATR; 1 and IL8, CYP1A1 and BRCA1 were not associated with any enriched pathway.

The associated proteins were further sorted based on a total number of enriched pathways. The median (which was 7) of all the numbers of the pathways covered by 22 possible key proteins was considered as a statistical cut-off, and by considering this cut-off, possible key regulatory proteins were sorted from 22 to 12 (MAPK1, AKT1, RELA, KRAS, TP53, NFKB1, SRC, RB1, CREBBP, STAT1, CREB1 and CASP8). These 12 possible key regulatory proteins were very crucial candidates of curcumin those have potential to efficiently interact with curcumin and regulate the whole curcumin-rewired PIN followed by induction of the desired pharmacological action. However, what will happen, while curcumin present in cell vicinity and which protein will hold the best binding affinity with curcumin, is not known. The answer to this question is molecular docking analysis, which is a globally accepted tool of biophysics and structural biology to calculate the binding energy. The aim of ligand–protein docking is to calculate the thermodynamic or binding affinity (ΔG; Gibbs free energy) and predominant binding mode(s) of a ligand with a protein. The docking results predicted that MAPK1/Erk2 (− 8.43 kcal/Mol), STAT1 (− 7.68Kcal/Mol) and KRAS (− 7.48Kcal/Mol) might be the best suitable targets of curcumin in its interactome (as shown in Supplementary Fig. 5), followed by P53 (− 6.57 kcal/Mol), CREBBP (− 6.29 kcal/Mol), RELA (− 6.07 kcal/Mol) and AKT1 (− 6.04 kcal/Mol), which also showed significant binding ability with curcumin. But when compared, the binding energies and the numbers of pathway coverage by these 12 proteins, then the most suitable and strongest candidate was MAPK1/Erk2 (highest binding energy − 8.43 kcal/Mol, and highest pathway coverage, i.e., 15) among other possible key proteins. Earlier published reports have also discussed the inhibition potential of curcumin on MAPK1/Erk2 and its activated and phosphorylated MAPK1/Erk2 entity^48,49^. Further, to endorse the above findings, a comparative docking analysis was performed between MAPK1/Erk2 and activated MAPK1/Erk2 with curcumin, and the result obtained showed that MAPK1/Erk2 (PDB Id: 3W55 & binding energy − 8.43 kcal/Mol, Ki 662.55 nM) binds more than twice strongly with curcumin compared to the activated MAPK1/Erk2 (PDB Id: 4IZA & binding energy − 4.15 kcal/Mol, Ki 910.81 µM). This result predicts that, if curcumin is present in the cell vicinity along with MAPK1/Erk2 and activated MAPK1/Erk2 then curcumin will have strong binding affinity towards MAPK1/Erk2 over activated MAPK/Erk2. This incidence designates that presence of curcumin on MAPK1/Erk2 induces some conformational changes in the protein skeleton, which would disturb the activation of MAPK1/Erk2 and block its activity. The molecular dynamics simulation was also strongly evident the strong and stable binding of MAPK1 and curcumin. Root mean square deviation calculations illustrated that the complex system of MAPK1-curcumin reached to a perfect equilibrium at around 25,000 picosecond and remains stable till the end of the simulation with very nominal fluctuations of 0.05 nm.

After getting strong evidences for MAPK1/Erk2 as a key regulatory protein, the bio-kinetics of MAPK cascade was simulated by using COPASI. In this experiment, bio-kinetics indicated the functional and concentration loss of MAPK1/Erk2 (from 0.0010782 µM/L to 0.000465626 µM/L in 150 s) and activated MAPK1/Erk2 (from 0.981202 µM/L to 0.0331032 µM/L in 150 s) in the presence of curcumin and alter the MAPK cascade, which may lead to several associated dysfunctions in the normal cell physiology like proliferation, differentiation and apoptosis. Overall, in depth topological, system protein–protein interactions, bio-kinetics and molecular dynamics analysis reveal that MAPK1 can be potentially considered as a key regulatory protein for inducing the desired pharmacological action of curcumin in the biological and cellular system.

## Conclusion

In this article, the anti-reductionism approach was implemented to illustrate the bigger picture of curcumin rewired biomolecular components system. Since, no reports have identified the complete topological network and key regulatory proteins of curcumin governed communication path yet, so in this article we have tracked the continuous development process of the research and tried to construct a possible curcumin associated PIN to the currently available information on curcumin. The key proteins were evaluated and obtained based on their topological parameters (degree, clustering coefficient and bottleneck scores), sub-modulization approach and functional ontology analyses. Finally, 12 most important key proteins (MAPK1/ERK2, AKT1, RELA, KRAS, TP53, NFKB1, SRC, RB1, CREBBP, STAT1, CREB1 and CASP8) were spotted, and found responsible to initiate and regulate curcumin governed pharmacological effects. Among all these key proteins MAPK1/ERK2 was identified as the most important key regulatory protein based on binding affinity and pathways enrichment analysis. MD conformational changes (MAPK1 on interaction with curcumin) and changes in protein concentration of MAPK cascade in the presence of curcumin via to simulate a biokinetic model also indicated the effect of curcumin on MAPK1 and its associated cascade. In conclusion, this study offers a competent way to explore the possible mechanistic actions of curcumin and opens the door for new clinical possibilities for novel drug development.

## Materials and methods

### Construction and visualization of protein–protein interaction network

788 altered biomolecular targets of curcumin were identified from 2,228 literatures by using PESCADOR text mining server. PESCADOR is a web-based tool to assist the text-mining of bio-interactions extracted from PubMed queries^15^, then further all the abstracts were manually-curated and standardized by authors.

The protein–protein interaction (PPI) network was obtained from the online databases of STRING 10.5 (https://string-db.org), which was used to retrieve the predicted interactions for the targets^16^. All associations available in STRING are provided with a probabilistic confidence score. The targets with a high confidence score greater than 0.7, experimental and curated databases were active interaction sources, max number of interactors in shell 1 and 2 were 50. These parameters were selected to construct the PPI network. Cytoscape software (version 3.6.1; https://www.cytoscape.org/), with a variety of network-related plugins were used for network visualization and integration platform ^17,18^.

### Protein interaction network (PIN) analysis

The topological properties are very important to bring an insight into the large complex networks^19,20^. In the procedure of protein interaction networks (PINs) analysis, Cytoscape and its in-build Network Analyzer plugin^21^ were used to calculate the basic topological parameters of the curcumin-rewired PINs, such as the degree distribution, clustering coefficient, betweeness centrality, closeness centrality, path distribution and topological coefficients distribution.

### Protein interaction network modular analysis and pathway enrichment

MCODE plugin of cytoscape was used for the modulation of big PINs. The term modulation stands for the find clusters (highly interconnected regions) in a network. Clusters mean different things in different types of networks. For example, clusters in a protein–protein interaction network are often protein complexes and parts of pathways, while clusters in a protein similarity network represent protein families^22^. Each module was scored by Cytoscape software through the density and size; a higher score meant a tighter module. Based on the identified modules, GO functional annotation and enrichment analysis were performed. Within the modules, pathway analyses were processed by the ClueGO plugin (version 2.5.1) according to the Kyoto Encyclopaedia of Genes and Genomes (KEGG) and Reactome database^23^, with a threshold of P < 0.05 based on a two-sided hypergeometric test and the Bonferroni correction were used.

### Molecular docking analysis

The aim of docking between ligand and biomolecule is, to predict the principal binding modes of a ligand with a biomolecule of known 3D structure. Docking studies were performed by Autodock 4.0 MGL suite^24,25^ in the Intel (R) i7-5500U, CPU 2.40 GHz and 16.0 GB of RAM of DELL Machine. The parameters used for docking studies using Autodock program for this study have been discussed in detail in our previously published article^26^. All the ten docking conformations of biomolecular targets and curcumin complex obtained were analysed for the interactions and binding energies of the docked structure using Discovery Studio visualizer version 3.1.

### Construction and simulation of bio-kinetic graph of key regulatory protein MAPK1/Erk2

The bio-kinetic model of MAPK cascade (BIOMD0000000009) was procured from BioModel Database (https://www.ebi.ac.uk/biomodels/). Cell Designer 4.4^27^ was used for the construction of new inhibited MAPK cascade (curcumin was used as an inhibitor) and COPASI^28^ was employed for the generation of rate kinetics and simulate bio-kinetic graph of both the reactions (normal and inhibited MAPK cascade).

### Molecular dynamics

MAPK1 docked complexes of curcumin was further subjected to molecular dynamics simulations using GROMACS version 5.0.7^29^. GROMACS compatible ligand parameter for curcumin were obtained from the Dundee PRODRG server^30^. A united atom force field, gromos53a6, was applied to the system solvated in space water molecules inside a triclinic box, which was neutralized using sodium and chloride ions. Energy minimization was done to achieve a maximum force of < 1,000.0 kJ/mol/nm with the steepest descent algorithm and verlet cut-off method. The system was then equilibrated up to 100 ps each at constant temperature (300 K) and pressure (1 bar), simultaneously^31–33^. All the bonds were constrained using LINCS algorithm and Particle Mesh Ewald (PME) method was used for long range electrostatics. The production run of molecular dynamics was performed for 50000 ps. All the post-MD analyses of the 50000 ps trajectory of MAPK1-curcumin complex were performed using the inbuilt scripts of GROMACS as done in earlier studies^34–36^.

## Supplementary information

Supplementary information
