# Supplementary material for: Topological and system-level protein interaction network (PIN) analyses to deduce molecular mechanism of curcumin
Source: Sci Rep. 2020 Jul 21;10:12045. doi: 10.1038/s41598-020-69011-0 (PMC7374742; doi:10.1038/s41598-020-69011-0)
Supplement: Supplementary file 1 — Supplementary information [file 41598_2020_69011_MOESM1_ESM.docx]

**Supplementary File**

**Topological and system-level protein interaction network (PIN) analyses to deduce molecular mechanism of curcumin**

Anupam Dhasmana^1,2^, Swati Uniyal^3^, Anukriti^2^, Vivek Kumar Kashyap^1^, Pallavi Somvanshi^4^, Meenu Gupta^2^, Uma Bhardwaj^2^, Meena Jaggi^1^, Murali M. Yallapu 1, Shafiul Haque^5^, Subhash C. Chauhan^1^

1) Department of Immunology and Microbiology, School of Medicine, University of Texas Rio Grande Valley, Edinburg, TX, USA

2) Department of Biosciences and Cancer Research Institute, Himalayan Institute of Medical Sciences, Swami Rama Himalayan University, Dehradun, India.

3) School of Biotechnology, Gautam Buddha University, Greater Noida, India.

4) Department of Biotechnology, TERI School of Advanced Studies, 10, Institutional Area, Vasant Kunj, New Delhi, India

5) Research and Scientific Studies Unit, College of Nursing and Allied Health Sciences, Jazan University, Jazan, Saudi Arabia

***Corresponding Author:** Prof. Subhash C. Chauhan Department of Immunology and Microbiology, School of Medicine, University of Texas Rio Grande Valley, Edinburg, TX, USA

**Competing interests**

The author(s) declare no competing interests.

**Supplementary Figures**

**
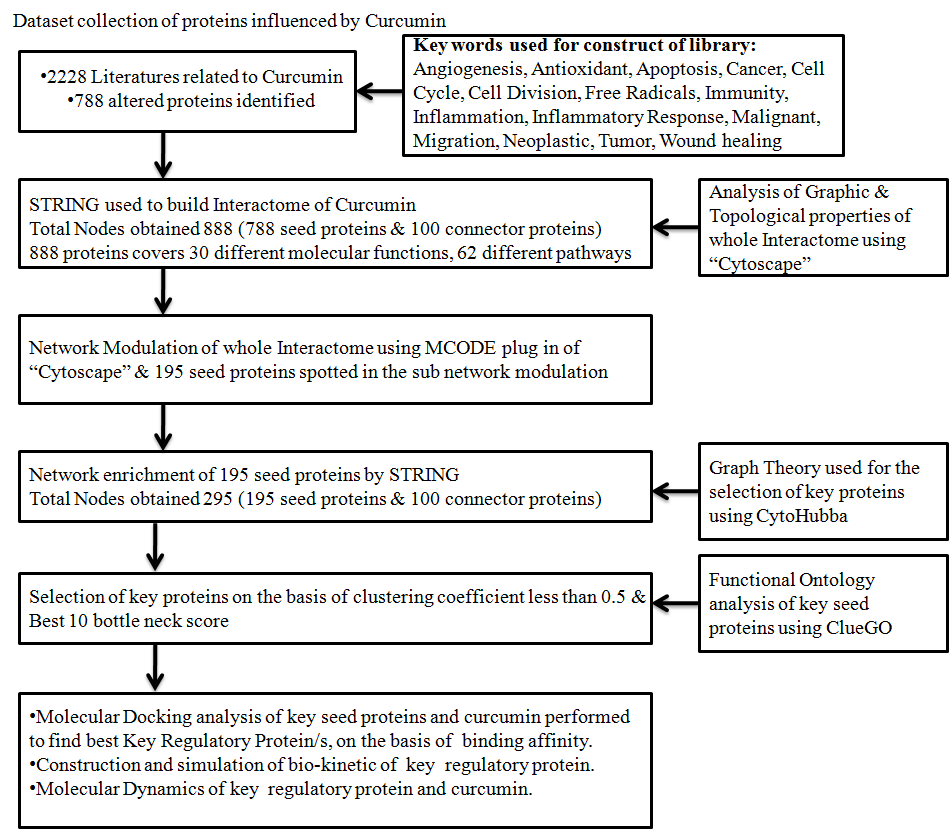
**

**Supplementary Figure 1:** Schematic diagram of protocol and analyses


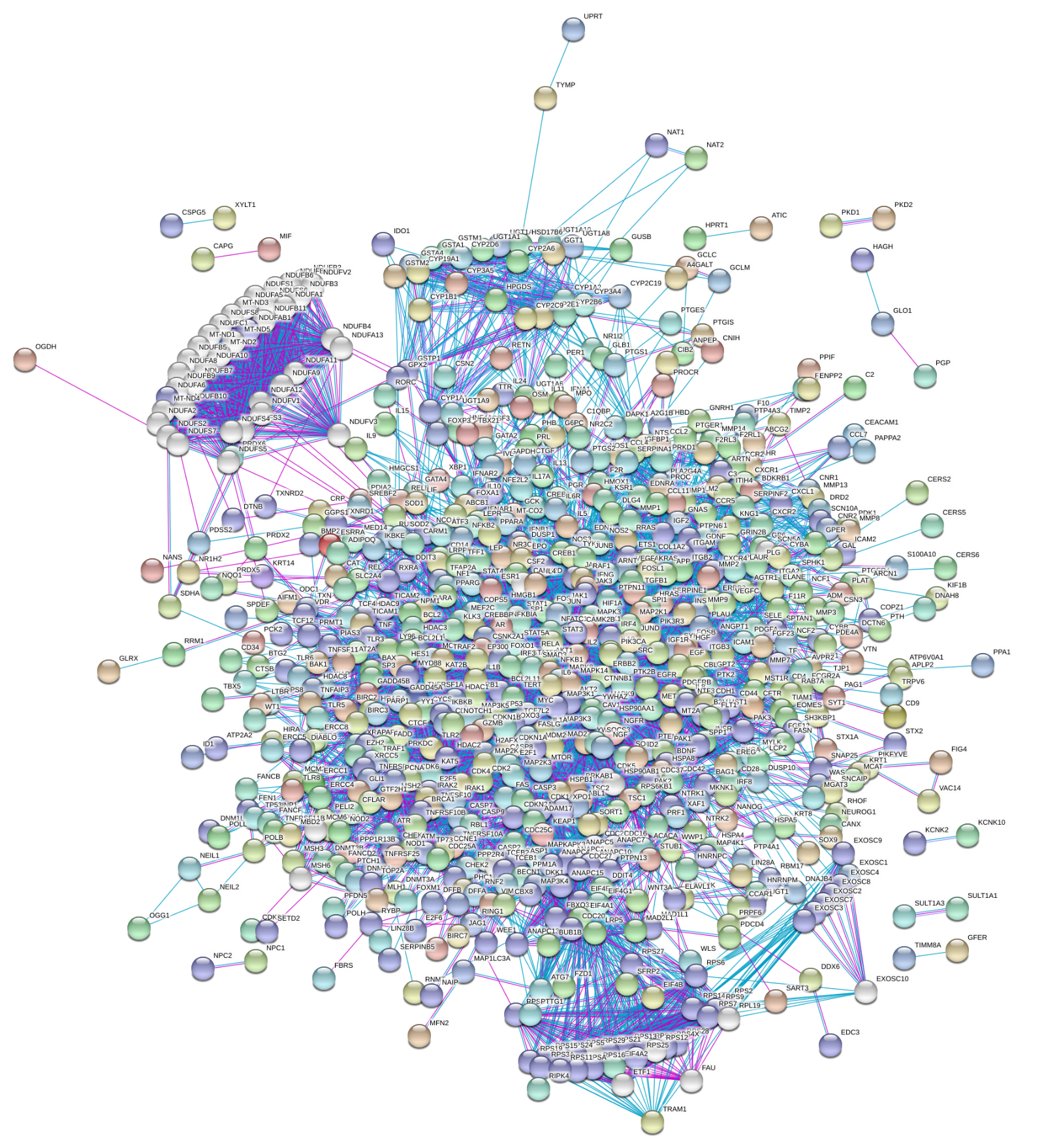


**Supplementary Figure 2:** The complete interactome (788 seed proteins & 100 connector proteins) of curcumin. Network was generated by STRING.


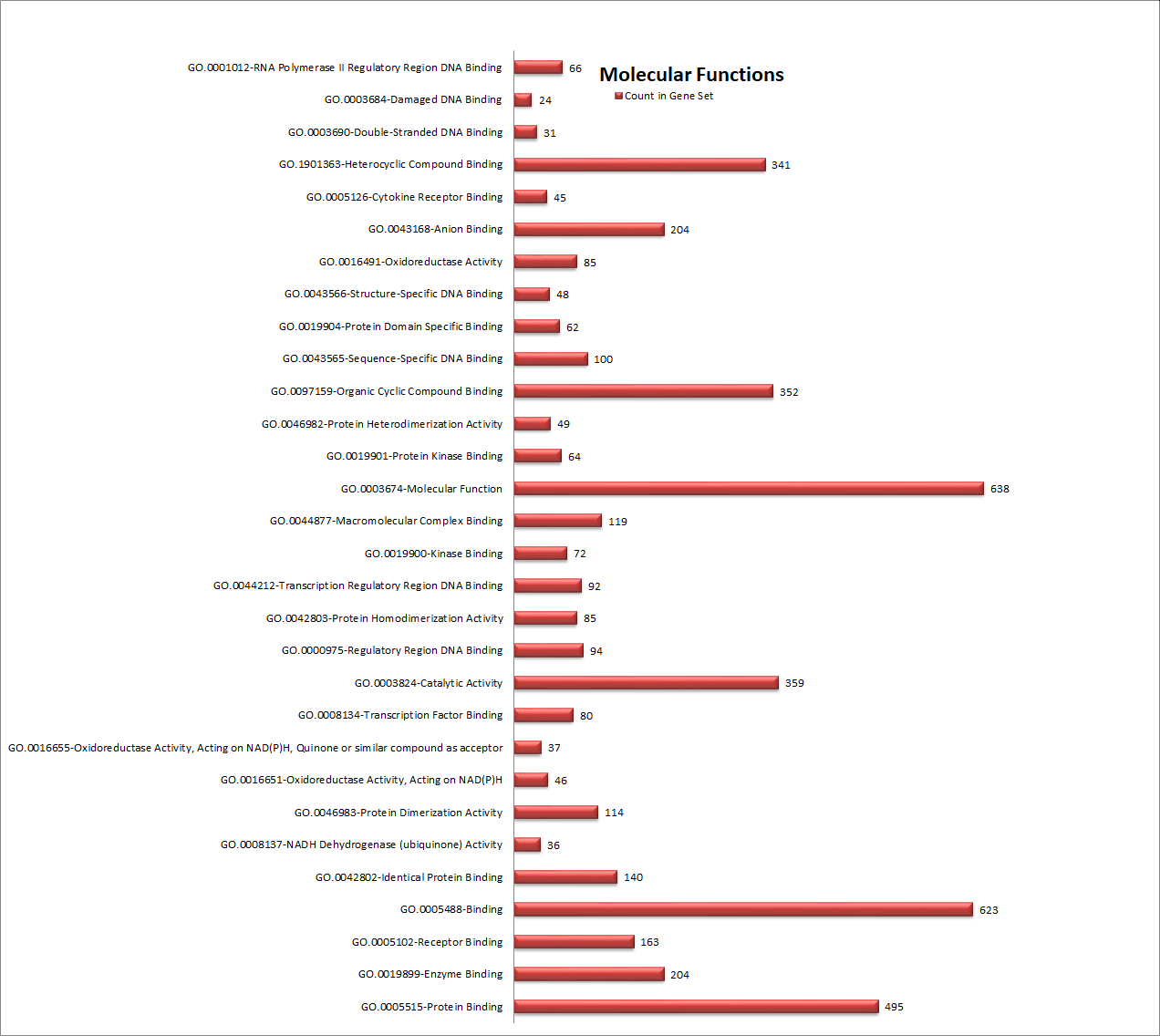


**Supplementary Figure 3:** Enrichment of 30 different molecular functions identified during the rewiring of curcumin associated proteins list of molecular function was generated by STRING


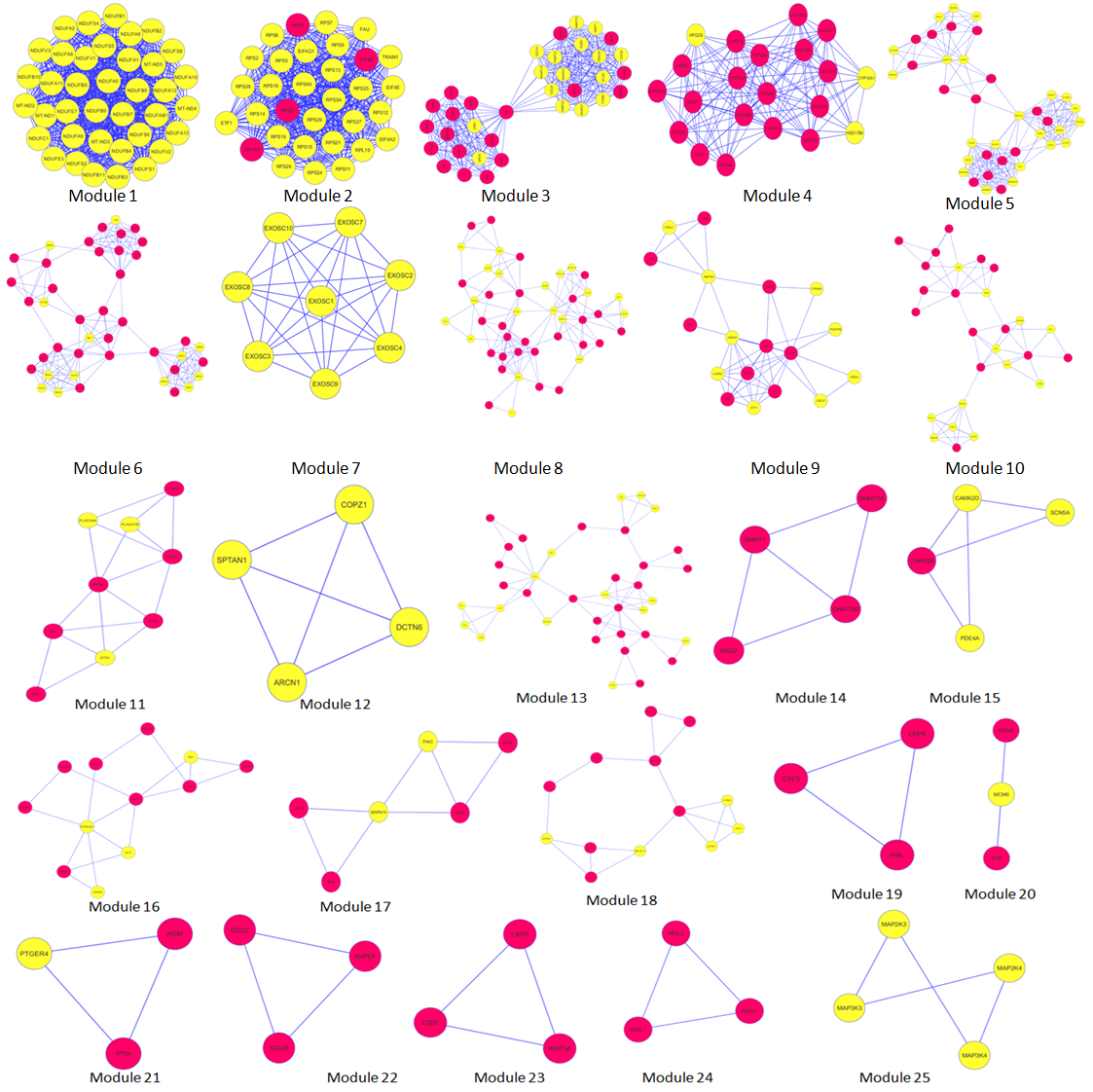


**Supplementary Figure 4:** Network modules: Critical modules with biological themes were identified from the total protein interaction network. Node colors represent seed proteins (red) or connecter proteins (yellow). Module 1, 7, 12, 25 (do not contains any seed proteins) and other yellow connector proteins will consider as noise.


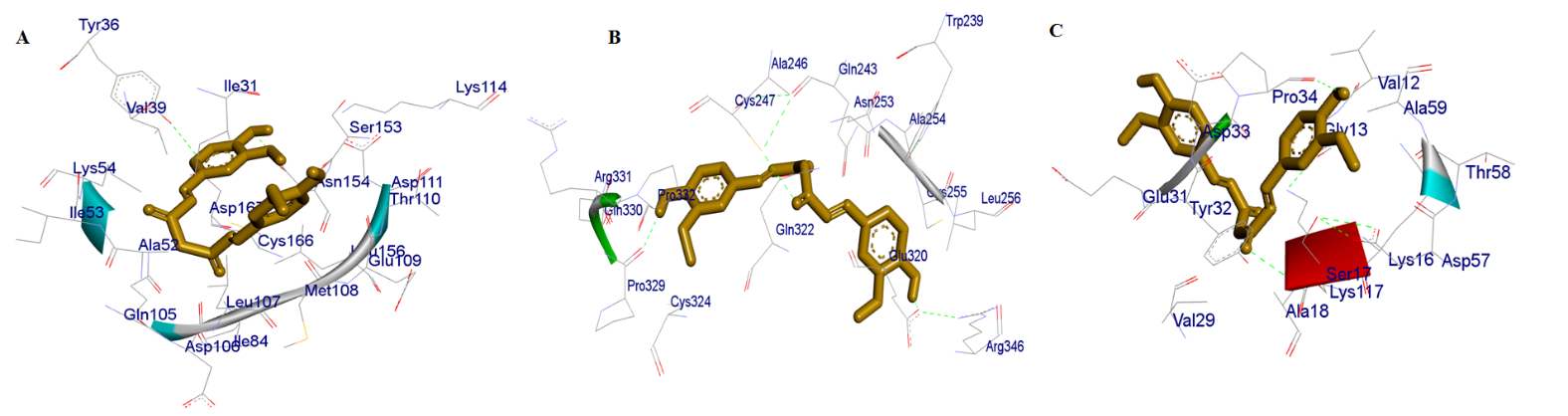


**Supplementary Figure 5:** (A) Binding interaction mode of Curcumin with MAPK1 (-8.43 Kcal/Mol); (B) Binding interaction mode of Curcumin with STAT1 (-7.68Kcal/Mol); (C) Binding interaction mode of Curcumin with KRAS (-7.48Kcal/Mol).

**Supplementary Table 1:** Network MCODE modules and enriched signalling pathways bold letters are seed proteins and unbold letters are connector proteins. Statistical Test Hypergeometric test, Significant p value 0.05

| **Cluster** | **MCODE Score** | **Nodes** | **Edges** | **Seed Proteins** | **Connecters** | **Node IDs** | **Pathway** | **P value**  **(Significant value 0.05)** |
| --- | --- | --- | --- | --- | --- | --- | --- | --- |
| 1 | 38 | 38 | 703 | 0 | 38 | MT-ND5, MT-ND1, NDUFS4, NDUFB3, NDUFB10, NDUFS5, NDUFB6, NDUFV2, NDUFB1, NDUFB9, NDUFB11, NDUFS6, NDUFA1, NDUFA11, NDUFA8, NDUFS8, NDUFS7, MT-ND3, NDUFA2, NDUFA5, MT-ND4, NDUFV3, NDUFB5, NDUFS1, NDUFB4, NDUFC1, NDUFA9, NDUFS3, NDUFV1, MT-ND2, NDUFAB1, NDUFB2, NDUFB7, NDUFS2, NDUFA12, NDUFA10, NDUFA6, NDUFA13 | Mitochondria electron Transport, NADH to ubiquinone  GO ID: 6120 | 4.4E-91 |
| 2 | 30.903 | 32 | 479 | 4 | 28 | RPL19, **EIF4A1,** RPS13, **RPS3,** RPS2, RPS15, RPS5, EIF4G1, EIF4E, RPS27, RPS11, RPS26, FAU, RPS9, ETF1, RPS14, EIF4A2, RPS7, RPS24, RPS19, **RPSA,** RPS25, RPS16, RPS21, RPS6, RPS3A, RPS12, RPS4X, TRAM1, **EIF4B,** RPS28, RPS29 | Nuclear Transcribed mRNA  Catabolic Process  GOID: 956 | 4.7E-59 |
| 3 | 17.588 | 35 | 299 | 19 | 16 | **DRD2,** ANAPC5, **GAL,ATG7,** ANAPC4, **CNR2,CDC27,** ANAPC2, **BDKRB1,** ANAPC7, ANAPC13, **CXCR2,KNG1,** WWP1, **KEAP1,** STUB1, **CXCR4,CXCL1,** ANAPC10, **CCR2,APP,CCR5,C3,** FBXO32, CDC16, GPER, **CXCR1,IL8,SOCS3,** TCEB2, TCEB1, CNR1, CDC23, **SOCS1,** CDC20 | 1) Positive Regulation of Protein Catabolic Process  GO ID: 45732  2) Protein Ubiqutation involved in ubiqutation dependent protein catabolic process  GO ID: 42787  3) Positive regulation of ubiqutin transferase activity  GO ID: 51443 | 1)4.9E-15  2) 8.2E-15  3)8.7E-15 |
| 4 | 14.4 | 21 | 144 | 18 | 3 | **GSTP1, UGT1A10, CYP1A2, CYP1A1, UGT1A8, GSTA1, GSTM2, GSTA4, GSTM1,** CYP19A1, HSD17B6, **UGT1A6, CYP2A6,** HPGDS, **CYP2C19, CYP2B6, UGT1A1, UGT1A7, CYP2C9, CYP3A4, UGT1A9** | 1. Xenobiotics Metabolic Process   GO ID: 6805  2) Cellular Response to Xenobiotic stimulus  GO ID: 71466  3) Response to Xenobiotic stimulus  GO ID: 9410 | 1) 1.5E-31  2) 2.7E-31  3) 8.0 E-31 |
| 5 | 10.158 | 39 | 193 | 16 | 23 | CDK2, F2RL1, CBX8, SERPINE1, PHC1, RNF2, SERPINA1, PLG, HGF, SERPINF2, NFKB1, MAD2L1, PTGER1, IL6, TIMP1, BRCA1, CDKN1B, NTS, ITIH4, CDKN1A, EDNRA, RELA, EDN1, GNRH1, F2R, CDK1, FN1, VEGFC, IGF2, ANAPC15, F2RL3, H2AFX, EGF, TGFB1, AGTR1, PIK3CA, PIK3R3, BUB1B, VEGFA | 1) Platelet Degranulations  GO ID: 2576 | 1) 2.8E-20 |
| 6 | 8.537 | 42 | 175 | 29 | 13 | TLR3, STAT5A, TICAM1, BIRC2, KAT2B, HDAC4, PRKDC, HDAC8, KAT2A, STAT1, MAPK3, ATM, CDK6, HDAC1, MSH3, HDAC3, HDAC9, ERCC8, HDAC2, IL6R, TLR4, E2F5, IKBKB, CD14, STAT4, STAT5B, NOTCH1, CASP8, RBL1, FADD, MSH2, MSH6, E2F1, LY96, ERCC1, ERCC4, STAT3, CDK4, TICAM2, XRCC6, CCNE1, XRCC5 | TRIF-dependent toll like receptor signaling pathway  GO ID: 35666 | 6.5E-20 |
| 7 | 8 | 8 | 28 | 0 | 8 | EXOSC1, EXOSC2, EXOSC8, EXOSC7, EXOSC3, EXOSC9, EXOSC4, EXOSC10 | 1) Nuclear m RNA surveillance  GO ID: 71028  2) Nuclear RNA surveillance  GO ID: 71027  3) RNA Surveillance  GO ID: 71025  4) Nuclear polyadenylation dependant nc RNA catabolic process  GO ID: 71046  5) Nuclear nc RNA surveillance  GO ID: 71029  6) Nuclear polyadenylation dependant r RNA catabolic process  GO ID: 71035 | 1)1.5E-22  2)1.5E-22  3) 5.6E-22  4) 7.6E-22  5) 7.6E-22  6) 7.6E-22 |
| 8 | 5.818 | 45 | 128 | 27 | 18 | NR3C1, TNFAIP3, ADIPOQ, YWHAQ, RXRA, YY1, MYD88, JAK2, NFATC1, CASP7, JAK1, JAK3, IL2, EZH2, BIRC3, TNFRSF1A, IL4, SLC2A4, IRF4, TNF, TRAF1, HMGB1, TYK2, IFNAR1, PPARG, CFLAR, TNFSF10, TNFRSF10B, APAF1, IFNG, DIABLO, FAS, PTPN11, INS, IKBKE, MED14, ITGB3, FASLG, NOS2, KSR1, TNFRSF10A, MAP3K5, RARA, FLT1, CASP9 | 1) Positive regulation of cysteine-type endopeptidase activity involved in apoptotic process  GO ID: 43280  2)Regulation of cysteine-type endopeptidase activity involved in apoptotic process  GO ID: 43281  3) Extrinsic apoptotic pathways  GO ID: 97191  4) Positive regulation of cysteine-type endopeptidase activity  GO ID: 2001056 | 1) 1.9E-19  2) 2.1E-19  3) 4.9E-19  4) 5.5E-19 |
| 9 | 5.647 | 18 | 48 | 9 | 9 | HSPA8, MAPK8, CDC37, **CD4,** CTNNB1, **CDC42, CFTR,** FOSL1, **CBL, EGFR, EPO, JUNB, JUND,** SYT1, **TF,** EREG, AVPR2, PDGFRB | 1) Negative regulation of ERBB Pathways  GO ID: 1901185  2) Positive regulation of DNA replication  GO ID: 45740 | 1) 2.7E-7  2) 4.1E-7 |
| 10 | 4.538 | 27 | 59 | 15 | 12 | CHEK2, MCL1, **CDH1,** PTK2, **ERBB2, SRC,** HNRNPC, **RBM17, HIF1A, KRAS, TERT,** CDKN2A, **ITGA2, IGF1R,** ITGB2, RB1, **ITGAM, BAK1, ERBB3, TP53, MAP2K1,** PRPF6, **ABL1,** KAT5, HNRNPM, ELAVL1, CCAR1 | 1) Cell Aging  GO ID:7569  2) Cellular Senscence  GO ID: 90398 | 1)2.E-10  2) 3.8.10 |
| 11 | 4.25 | 9 | 17 | 6 | 3 | PLA2G4A, PLA2G1B, **ARNT,** ETS1, **FOS, PTGS1**, **SP1, PTGS2**, **MAPK1** | 1) Icosanoid biosynthetic process  GO ID:46546 | 1)1.2E-8 |
| 12 | 4 | 4 | 6 | 0 | 4 | ARCN1, SPTAN1, DCTN6, COPZ1 |  |  |
| 13 | 3.778 | 37 | 68 | 22 | 15 | **PAK1,** ERCC5, LTBR, **FGF23,RELB,FOXO1,MET,PDGFA,CTGF,** PARP1, TNFSF11, **TRAF2,AR,TIAM1,** TNFRSF1B, NCOA2, **NFKB2,** HRAS, **FANCD2,SPHK1,SMAD2,SMAD3,** CYBA, NCF1, NCF2, **NFKBIA,PPARA,** HSP90AB1, TXNRD1, **CREBBP,HES1,PCNA,RAF1,** FANCB, **XIAP,** HMGCS1, CYBB | 1) Positive Regulation of JAK cascade  GO ID: 46330  2) Regulation of stress activated MAPK cascade  GO ID:32872  3) Regulation of stress activated Protein Kinases Signalling Cascade.  GO ID: 70302  4) Positive regulation of stress activated MAPK cascade  GO ID:32874  5) Positive Regulation of stress activated Protein Kinases Signalling Cascade.  GO ID: 70304 | 1) 1.6E-10  2) 4.6E-10  3)4.8E-10  4) 5.9E-10  5 ) 6.2E-10 |
| 14 | 3.333 | 4 | 5 | 4 | 0 | **DNMT1, DNMT3B, MBD2, DNMT3A** | C5 methylation of cytosine  GO ID: 90116 | 4.6E-12 |
| 15 | 3.333 | 4 | 5 | 1 | 3 | **CAMK2B,** PDE4A, SCN5A, CAMK2D | Regulation of muscle adaptation  GO ID: 43502 | 3.1E-7 |
| 16 | 3.273 | 12 | 18 | 8 | 4 | **DDIT4, WEE1, HSPA4,** HSPA5, HSP90AA1, **AKT1,** TSC1, **YWHAZ, CREB1, DUSP1,** MYC, **CDC25C** | 1) Responses to nerve growth factor  GO ID: 1990089  2) Cellular Responses to nerve growth factor  Stimulus  GO ID: 1990090 | 1)4.6E-6  2)3.8E-6 |
| 17 | 3.2 | 6 | 8 | 4 | 2 | **SYK,** PAK3, **PAK2,** MAPK14, **MYLK, JUN** |  |  |
| 18 | 3.167 | 13 | 19 | 8 | 5 | NTRK2, NTF3, **NGF,** NTRK1, BCL2L11, **IRF3, MMP13, BCL2,** EP300, **CD44, BAX, MMP7, MMP3** | 1) Negative regulation of neuron apoptotic process  GO ID: 43524 | 1) 5.6E-10 |
| 19 | 3 | 3 | 3 | 3 | 0 | **PRL, CSF3, LEPR** |  |  |
| 20 | 3 | 3 | 3 | 2 | 1 | MCM6, **ATR, MCM2** |  |  |
| 21 | 3 | 3 | 3 | 2 | 1 | **ADM,** PTGER4, **PTH** |  |  |
| 22 | 3 | 3 | 3 | 3 | 0 | **GCLC, GCLM, ANPEP** |  |  |
| 23 | 3 | 3 | 3 | 3 | 0 | **WNT3A, FZD1, LRP5** | Regulation of muscle adaptation  GO ID: 43502 | 3.1E-7 |
| 24 | 3 | 3 | 3 | 3 | 0 | **OGG1, NEIL1, NEIL2** | Depyrimidation  GO ID: 45008 | 6.5E-11 |
| 25 | 2.667 | 4 | 4 | 0 | 4 | MAP3K4, MAP2K3, MAP3K3, MAP2K4 |  |  |

**Supplementary Table 1b:** Description of 195 seed proteins with their protein ID, Pubmed ID and impact of curcumin.

| **S.No.** | **Protein Name** | **Protein ID/Uniprot ID** | **Pubmed ID** | **Impact** |
| --- | --- | --- | --- | --- |
| 1 | CFTR | [P13569](https://www.uniprot.org/uniprot/P13569) | [15582996](http://www.ncbi.nlm.nih.gov/pubmed/15582996) | Curcumin results in increased activity of CFTR protein |
| 2 | CD4 | P01730 | 23658623 | Curcumin inhibits CD4 T cell activation |
| 3 | ERCC1 | [P07992](https://www.uniprot.org/uniprot/P07992) | [21493726](http://www.ncbi.nlm.nih.gov/pubmed/21493726) | Curcumin results in decreased expression of ERCC1 mRNA |
| 4 | MAPK1 | [P28482](https://www.uniprot.org/uniprot/P28482) | [21493726](http://www.ncbi.nlm.nih.gov/pubmed/21493726) | Curcumin results in decreased activity of MAPK1 |
| 5 | NFKBIA | [P25963](https://www.uniprot.org/uniprot/P25963) | [16023083](http://www.ncbi.nlm.nih.gov/pubmed/16023083) | Curcumin results in decreased expression of NFKBIA protein |
| 6 | RELB | Q01201 | [21070208](https://www.ncbi.nlm.nih.gov/pubmed/21070208) | CurcDC also displayed decreased RelB and interleukin (IL)-12 mRNA and protein expression. |
| 7 | HGF | P08581 | 10713700 | HGF is inhibited by the addition of Curcumin |
| 8 | CSF3 | [P09919](https://www.uniprot.org/uniprot/P09919) | 19723087 | we investigated the effect of curcumin pretreatment on 84 tumor necrosis factor-alpha (TNF-alpha)-activated genes of NF-kappaB pathways in K562 cells, using a real-time PCR array. Our results show that transcription of 29 NF-kappaB-related mRNAs was significantly downregulated (CARD4, CCL2, CD40, CSF2, F2R, ICAM1, IKBKB, IKBKE, IL1A, IL1B, IL6, IL8, IRAK2, MALT1, MAP3K1, MYD88, NFKB1, NFKB2, NFKBIA, PPM1A, RAF1, RELB, STAT1, TLR3, TNF, TNFalphaIP3, TNFSF10, and TICAM1), whereas 10 mRNAs were induced (AGT, CASP1, CSF3, FOS, IFNG, IL10, TICAM2, TLR2, TLR9, and TNFRSF7). |
| 9 | NFKB1 | [P19838](https://www.uniprot.org/uniprot/P19838) | 16173963, 17041101, 17596214 | Curcumin results in decreased activity of NFKB1 protein |
| 10 | IL2 | [P60568](https://www.uniprot.org/uniprot/P60568) | [19761891](http://www.ncbi.nlm.nih.gov/pubmed/19761891) | Curcumin results in decreased expression of and results in decreased secretion of IL2 protein |
| 11 | BIRC2 | [Q13490](https://www.uniprot.org/uniprot/Q13490) | [17671737](http://www.ncbi.nlm.nih.gov/pubmed/17671737) | Curcumin analog results in decreased expression of BIRC2 mRNA |
| 12 | IFNG | [P01579](https://www.uniprot.org/uniprot/P01579) | [16959222](http://www.ncbi.nlm.nih.gov/pubmed/16959222) | Curcumin inhibits the reaction [IFNG protein results in increased expression of and results in increased phosphorylation of STAT1 protein] |
| 13 | GCLC | [P48506](https://www.uniprot.org/uniprot/P48506) | [10514034](http://www.ncbi.nlm.nih.gov/pubmed/10514034) | Curcumin results in increased activity of GCLC protein |
| 14 | IL4 | [P05112](https://www.uniprot.org/uniprot/P05112) | 24211923 | Curcumin increase the level of IL4 activity of GCLC protein |
| 15 | NR3C1 | [P04150](https://www.uniprot.org/uniprot/P04150) | [18483179](http://www.ncbi.nlm.nih.gov/pubmed/18483179) | Curcumin inhibits the reaction [Dexamethasone results in increased phosphorylation of NR3C1 protein] |
| 16 | HES1 | Q14469 | 24024180 | Notch1 and its target genes (Hes1 and cyclin D1) were downregulated in all treated groups with more suppressive effect in the groups treated with both MSCs and NCD. |
| 17 | MSH2 | P43246 | 12973926 | After irradiation and addition of curcumin, the expression of hMSH2 mRNA increased and the cellular apoptotic rate also increased at the same time |
| 18 | FGF23 | [Q9GZV9](https://www.uniprot.org/uniprot/Q9GZV9) | 27967217 | short-term curcumin gavage stimulated mouse hepatic fibroblast growth factor 21 (Fgf21) expression |
| 19 | DUSP1 | [P28562](https://www.uniprot.org/uniprot/P28562) | [15713895](http://www.ncbi.nlm.nih.gov/pubmed/15713895) | Curcumin results in increased expression of DUSP1 mRNA |
| 20 | TNFSF10 | [P50591](https://www.uniprot.org/uniprot/P50591) | 16101141, 15713895 | Curcumin results in decreased expression of TNFSF10 mRNA |
| 21 | GSTM2 | P28161 | 29887802 | Transcription factor Nrf2, its downstream genes such as GSTA3, and GSTM2 mRNA, and protein expression level significantly upregulated via dietary curcumin |
| 22 | C3 | P01024 | 23410788 | Curcumin increased IgG deposits and decreased C3 deposits in brain with a corresponding increase in immune complexes and decrease in C3 concentration (by 60% in MRL/lpr mice Vs. |
| 23 | TRAF2 | [Q12933](https://www.uniprot.org/uniprot/Q12933) | [16101141](http://www.ncbi.nlm.nih.gov/pubmed/16101141) | Curcumin results in increased expression of TRAF2 mRNA |
| 24 | TICAM1 | [Q8IUC6](https://www.uniprot.org/uniprot/Q8IUC6) | 19723087 | Curcumin results in decreased expression of TICAM1 |
| 25 | INS | P01308 | [30209353](https://www.ncbi.nlm.nih.gov/pubmed/30209353) | Curcumin results in decreased demand of Insulin (INS) |
| 26 | RAF1 | P04049 | 16101141 | Curcumin results in upregulate Craf1 |
| 27 | EPO | [P01588](https://www.uniprot.org/uniprot/P01588) | [16880289](http://www.ncbi.nlm.nih.gov/pubmed/16880289) | Curcumin results in decreased expression of EPO mRNA |
| 28 | JUND | [P17535](https://www.uniprot.org/uniprot/P17535) | [17148446](http://www.ncbi.nlm.nih.gov/pubmed/17148446) | Curcumin inhibits the reaction |
| 29 | KRAS | [P01116](http://www.ncbi.nlm.nih.gov/pubmed/17041101) | 17041101 | Curcumin analog results in decreased expression of KRAS mRNA |
| 30 | MBD2 | [Q9UBB5](https://www.uniprot.org/uniprot/Q9UBB5) | 21938566 | CUR treatment had limited effects on the expression of epigenetic modifying proteins MBD2 |
| 31 | CDK4 | P11802 | [18156803](http://www.ncbi.nlm.nih.gov/pubmed/18156803) | Curcumin results in decreased expression of CDK4 protein |
| 32 | IL6 | [P05231](https://www.uniprot.org/uniprot/P05231) | [19074641](http://www.ncbi.nlm.nih.gov/pubmed/19074641) | Curcumin inhibits the reaction [Acids results in increased expression of IL6 mRNA] |
| 33 | MMP7 | [P09237](https://www.uniprot.org/uniprot/P09237) | [17928719](http://www.ncbi.nlm.nih.gov/pubmed/17928719) | Curcumin inhibits the reaction [epigallocatechin gallate results in increased expression of MMP7 protein] |
| 34 | MMP13 | [P45452](https://www.uniprot.org/uniprot/P45452) | [20338993](http://www.ncbi.nlm.nih.gov/pubmed/20338993) | Curcumin inhibits the reaction [Chloramphenicol results in increased expression of MMP13 mRNA] |
| 35 | CYP2C9 | P11712 | 22725836 | Previous in vitro studies indicate that curcuminoids could inhibit CYP2C9 dependent drug metabolism. |
| 36 | CDH1 | [P12830](https://www.uniprot.org/uniprot/P12830) | [19573523](http://www.ncbi.nlm.nih.gov/pubmed/19573523) | Curcumin results in increased expression of CDH1 protein |
| 37 | EIF4B | P23588 | 20145189 | In the immortalized, leukoplakia, and CANCER cells, curcumin inhibited cap-dependent translation by suppressing the phosphorylation of 4E-BP1, eIF4G, eIF4B, and Mnk1, and also reduced the total levels of eIF4E and Mnk1. |
| 38 | SMAD2 | [Q15796](https://www.uniprot.org/uniprot/Q15796) | 22978413 | curcumin significantly attenuated expressions of TGFβ1, Smad2 |
| 39 | YY1 | [P25490](https://www.uniprot.org/uniprot/P25490) | 18794131 | The novel candidate target of curcumin is HLJ1, and endogenous control of HLJ1 transcription in cancer cells is regulated by transcriptional factor YY1 and AP-1. |
| 40 | CREBBP | [Q92793](https://www.uniprot.org/uniprot/Q92793) | [22258452](http://www.ncbi.nlm.nih.gov/pubmed/22258452) | Curcumin inhibits the reaction [Dihydrotestosterone promotes the reaction [CREBBP protein binds to KLK3 enhancer]] |
| 41 | CCNE1 | [P24864](https://www.uniprot.org/uniprot/P24864) | [17148446](http://www.ncbi.nlm.nih.gov/pubmed/17148446) | Curcumin results in decreased expression of CCNE1 protein |
| 42 | PPARA | Q07869 | 16713233 | Expression of the regulatory SREBP genes was moderately increased, whereas mRNAs of the PPARalpha target genes CD36/fatty acid translocase and fatty acid binding protein 1 were down-regulated. |
| 43 | BIRC3 | [Q13489](https://www.uniprot.org/uniprot/Q13489) | [17596214](http://www.ncbi.nlm.nih.gov/pubmed/17596214) | Curcumin results in decreased expression of BIRC3 mRNA |
| 44 | CBL | P22681 | 22849866 | Curcumin (Cur), demethoxycurcumin (DMC), and bisdemethoxycurcumin (BDMC) are major forms of curcuminoids found in the rhizomes of turmeric. DMC also increased the interaction between EGFR and CBL. |
| 45 | STAT3 | [P40763](http://www.ncbi.nlm.nih.gov/pubmed/20816778) | 20816778 | Curcumin results in decreased expression of STAT3 mRNA |
| 46 | DNMT3A | Q9Y6K1 | 21938566 | At the protein level, CUR treatment had limited effects on the expression of epigenetic modifying proteins MBD2, MeCP2, DNMT1, and DNMT3. |
| 47 | TYK2 | P29597 | 16364242 | Inhibition of JAK3 and TYK2 phosphorylation by curcumin |
| 48 | MCM2 | P49736 | 22156994 | Curcumin reduced MCM2 in mice lung cancer |
| 49 | EGF | [P01133](https://www.uniprot.org/uniprot/P01133) | [18214481](http://www.ncbi.nlm.nih.gov/pubmed/18214481) | Curcumin inhibits the reaction |
| 50 | GALN | P22466 | 19856147 | Inhibit the effect of GALN |
| 51 | CDK6 | Q00534 | 20686221 | Curcumin inhibited the expression of CDK6 |
| 52 | CXCL8 | P10145 | 24276377 | The ability of curcumin to inhibit the synthesis and signaling of CXCL8 |
| 53 | CDK2 | P24941 | 20686221 | Curcumin inhibited the expression of CDK2 |
| 54 | ERBB3 | P21860 | [17918158](http://www.ncbi.nlm.nih.gov/pubmed/17918158) | Curcumin results in decreased expression of and results in decreased phosphorylation of and results in decreased activity of ERBB3 protein |
| 55 | IGF1R | P08069 | [17499312](http://www.ncbi.nlm.nih.gov/pubmed/17499312) | Curcumin results in decreased expression of IGF1R mRNA |
| 56 | TP53 | P04637 | 24896104 | curcumin treatment up-regulated the expression of TP53. |
| 57 | ERBB2 | P04626 | 17239458 | curcumin induced degradation of ERBB2 |
| 58 | IFNAR1 | P17181 | 17979888 | Curcumin increased IFN-alpha-induced IL-10 and IFNAR1 expression. |
| 59 | AKT1 | P31749 | 31545905 | incubation with Curcumin decreased AKT1 and p-AKT1(Thr308) levels |
| 60 | EGFR | P00533 | [10851300](http://www.ncbi.nlm.nih.gov/pubmed/10851300) | curcumin inhibits the phosphorylation of EGFR |
| 61 | NOTCH1 | P46531 | 24024180 | NOTCH1 was downregulated by curcumin. |
| 62 | ADM (Adrenomedullin) | P35318 | 26002528 | Curcumin may potentiate Adrenomedullin. In a murine model of virally induced ARDS, pretreatment with curcumin significantly reduced a number of surrogate severity markers of ARDS and Adrenomedullin, a vasoactive peptide hormone initially isolated from pheochromocytoma tissue has been gaining interest as a potential biomarker for ARDS and may have therapeutic potential. |
| 63 | PAK1 | Q13153 | 19448398 | curcumin inhibited the kinase activity of PAK1 |
| 64 | ATM | Q13315 | [19401701](http://www.ncbi.nlm.nih.gov/pubmed/19401701) | Curcumin results in increased phosphorylation of ATM protein |
| 65 | PTH | P01270 | 17541507 | curcumin inhibits the activity of PTH |
| 66 | NEIL2 | Q969S2 | 20622253 | curcumin, reverse the inhibition of NEILs both in vitro and in cells |
| 67 | WNT3A | P56704 | 31602860 | curcumin downregulates the expression of Wnt3a |
| 68 | TIAM1 | Q13009 | 14637190, 18347024 | Curcumin has inhibitory effect ov SRC which phosphorylates TIAM1 |
| 69 | FANCD2 | Q9BXW9 | 19756599 | curcumin downregulates the ubiquitination of FANCD2 protein |
| 70 | PPARG | P37231 | 19297423 | curcumin significantly lowers the expression of PPARG |
| 71 | FZD1 | Q9UP38 | 15713895 | curcumin represses the expression of FZD1 |
| 72 | CCR5 | P51681 | 15569263 | curcumin inhibited Abeta1-40-induced expression of CCR5 |
| 73 | BAX | Q07812 | 24083709 | curcumin upregulates the expression of Bax |
| 74 | EIF4A1 | P60842 | 20145189 | curcumin suppresses the phosphorylation of eIF4B which ultimately supresses the activity of EIF4A |
| 75 | LRP5 | O75197 | 26300394 | Treatment of SCI rats with curcumin enhanced mRNA levels of Wnt3a, Lrp5, and ctnnb1 and upregulated protein expression of β-catenin in distal femurs. |
| 76 | CXCR1 | P25024 | [12216086](http://www.ncbi.nlm.nih.gov/pubmed/12216086) | Curcumin results in increased expression of CXCR1 protein |
| 77 | MAD2L1 | Q13257 | 20646066 | curcumin increased the accumulation of Mad2 at the kinetochores |
| 78 | ITGA2B | P17301 | 28618934 | significantly inhibited by curcumin |
| 79 | TLR3 | O15455 | 19723087 | Curcumin inhibits the transcription of TLR3 |
| 80 | MMP3 | P08254 | 15713895,  18321735 | Curcumin results in increased expression of MMP3 mRNA |
| 81 | ANPEP | P15144 | 12954328 | Curcumin irreversibly inhibits aminopeptidase N |
| 82 | CYP2A6 | P11509 | 28377720 | curcumin inhibits CYP2A6 |
| 83 | FADD | Q13158 | 19513510 | Curcumin increased the levels of FADD |
| 84 | PRL | P01236 | 19726538 | In endocrine pituitary tumor curcumin inhibited the production of prolactin. |
| 85 | MAP2K1 | Q02750 | 21973306 | Curcumin decreases the phosphorylation of MAP2K1 |
| 86 | HSPA4 | P34932 | [11322385](http://www.ncbi.nlm.nih.gov/pubmed/11322385) | Curcumin results in increased expression of HSPA4 mRNA |
| 87 | HDAC3 | O15379 | 21938566 | curcumin decreased the expression of HDAC3 |
| 88 | UGT1A6 | P19224 | [16819192](http://www.ncbi.nlm.nih.gov/pubmed/16819192) | Curcumin results in increased expression of UGT1A6 mRNA |
| 89 | CD14 | P08571 | [18180316](http://www.ncbi.nlm.nih.gov/pubmed/18180316) | curcumin inhibits the expression of CD14 |
| 90 | UGT1A1 | P22309 | [16819192](http://www.ncbi.nlm.nih.gov/pubmed/16819192) | Curcumin results in increased expression of UGT1A1 mRNA |
| 91 | FOS | P01100 | [17999991](http://www.ncbi.nlm.nih.gov/pubmed/17999991) | Curcumin results in decreased expression of FOS mRNA |
| 92 | IL8 | [P10145](https://www.uniprot.org/uniprot/P10145) | [19074641](http://www.ncbi.nlm.nih.gov/pubmed/19074641) | Curcumin inhibits the reaction [Acids results in increased expression of IL8 mRNA] |
| 93 | OGG1 | O15527 | 21332098 | Curcumin suppressed OGG1 expression |
| 94 | DDIT4 | Q9NX09 | 26238775 | curcumin downregulates the expression of DDIT4 |
| 95 | YWHAZ | P63104 | 17324259 | Increase the mRNA expression level in higher conc. |
| 96 | TERT | O14746 | 20363232 | curcumin decreases TERT |
| 97 | IRF3 | Q14653 | 16678799 | Curcumin inhibited LPS-induced IRF3 activation. |
| 98 | ERCC4 | Q92889 | 22228707 | curcumin helps in reduction of ERCC4 levels |
| 99 | GSTM1 | P09488 | 19350453 | Curcumin inhibits GSTM1 |
| 100 | PRKDC | P78527 | [17596214](http://www.ncbi.nlm.nih.gov/pubmed/17596214) | Curcumin results in decreased expression of PRKDC mRNA |
| 101 | SPHK1 | Q9NYA1 | 23127801 | curcumin downregulated the activity of SPHK1 |
| 102 | PAK2 | Q13177 | 16624471 | curcumin cleaves PAK2 |
| 103 | CDC42 | P60953 | 22266952 | curcumin modulates the expression of CDC42 |
| 104 | MET | P08581 | 10713700 | curcumin inhibits the induction of endogenous c-met gene by HGF |
| 105 | EZH2 | Q15910 | 22952749 | curcumin down-regulates the expression of EZH2 mRNA |
| 106 | F2R | P25116 | 19723087 | curcumin down-regulates the transcription of F2R |
| 107 | CDC25C | P30307 | [19401701](http://www.ncbi.nlm.nih.gov/pubmed/19401701) | Curcumin results in increased phosphorylation of CDC25C protein |
| 108 | MED14 | O60244 | 18483179 | S211 phosphorylation enhances GR interaction with MED14 (vitamin D receptor interacting protein 150) and Curcumin, supresses NFκB pathway through an unknown mechanism, inhibited GR phosphorylation at S211 at the highest concentration tested (50 μm). |
| 109 | CYP2B6 | P20813 | 18480186 | curcuminoid extract inhibited the activity of CYP2B6 |
| 110 | NOS2 | P35228 | [17640567](http://www.ncbi.nlm.nih.gov/pubmed/17640567) | Curcumin inhibits the reaction [TNF protein results in increased expression of NOS2 protein] |
| 111 | DNMT3B | Q9UBC3 | 24138392 | DNA methyltransferase 3b (DNMT3b) was reduced in vivo and in vitro after curcumin treatment |
| 112 | SP1 | P08047 | 23457487 | curcumin reduced the expression of the positive regulator of Sp1 |
| 113 | SOCS1 | O15524 | 23430957 | pure curcumin increased the expression of SOCS1 |
| 114 | BCL2 | P10415 | 17671737 19250217 | Curcumin analog results in decreased expression of BCL2 mRNA |
| 115 | CASP9 | P55211 | [17148446](http://www.ncbi.nlm.nih.gov/pubmed/17148446) | Curcumin results in decreased expression of CASP9 protein |
| 116 | SOCS3 | O14543 | 23430957 | pure curcumin increased the expression of SOCS3 |
| 117 | LEPR | P48357 | 30057682 | Curcumin interrupts leptin signaling by reducing phosphorylation levels of leptin receptor (Ob-R) |
| 118 | SMAD3 | P84022 | 23609161 | curcumin inhibits the phosphorylation of SMAD3 |
| 119 | GSTA1 | P08263 | 19350453 | curcumin inhibits GSTA1 |
| 120 | CYP3A4 | P08684 | 22512082 | curcumin inhibits CYP3A4 |
| 121 | HIF1A | Q16665 | 22739211 | curcumin suppresses the synthesis of HIF1A |
| 122 | STAT5A | P42229 | 16959222 | curcumin treatment decreased STAT5A |
| 123 | CYP1A2 | P05177 | 20484172 | curcumin affects the activity of CYP1A2 |
| 124 | HMGB1 | P09429 | 28929026 | Curcumin suppresses the release of HMGB1 |
| 125 | JAK1 | P23458 | 16044161 | curcumin acts as a supressor for activation of JAK1 |
| 126 | ATR | Q13535 | 23825154 | curcumin inhibits ATR |
| 127 | UGT1A10 | Q9HAW8 | [14557274](http://www.ncbi.nlm.nih.gov/pubmed/14557274) | UGT1A10 protein results in increased glucuronidation of Curcumin |
| 128 | E2F1 | Q01094 | 23192708 | curcumin down-regulates E2F1 |
| 129 | RPSA | P08865 | 22315092 | RPSA is significantly modulated in SH-SY5Y cells sensitive to cisplatin (WT) after 40 mM curcumin treatment identified by label-free LC-MSE |
| 130 | ATG7 | O95352 | 22622204 | under oxidative stress condition, curcumin elevates the level of interaction of acetylated ATG7 |
| 131 | PDGFA/PDGF1 | P04085 | 16239599 | Curcumin inhibits platelet-derived growth factor (PDGF)-stimulated vascular smooth muscle cell function and injury-induced neointima formation. |
| 132 | UGT1A9 | O60656 | [14557274](http://www.ncbi.nlm.nih.gov/pubmed/14557274) | UGT1A9 protein results in increased glucuronidation of Curcumin |
| 133 | FN1 | P02751 | [18555241](http://www.ncbi.nlm.nih.gov/pubmed/18555241) | Curcumin results in decreased expression of FN1 mRNA |
| 134 | NEIL1 | Q96FI4 | [20622253](http://www.ncbi.nlm.nih.gov/pubmed/20622253) | Curcumin inhibits the reaction [Copper binds to and results in decreased activity of NEIL1 protein] |
| 135 | XIAP | P98170 | 20651361 | curcumin down-regulated XIAP |
| 136 | FAS | P25445 | 19513510 | curcumin promoted the levels of FAS |
| 137 | SRC | P12931 | 19779032 | curcumin inhibited the phosphorylation of SRC |
| 138 | STAT4 | Q14765 | 17979888 | curcumin activates STAT4 |
| 139 | CASP8 | Q14790 | [17148446](http://www.ncbi.nlm.nih.gov/pubmed/17148446) | Curcumin results in decreased expression of CASP8 protein |
| 140 | ARNT | P27540 | [19018768](http://www.ncbi.nlm.nih.gov/pubmed/19018768) | Curcumin results in increased degradation of ARNT protein |
| 141 | MAP3K5 | Q99683 | 22875542 | We found that the largest proportion of genes involved in curcumin inhibition of renal inflammation induced by LPS belong to those in the MAPK signaling pathway, including the genes Crk II, Rras2, Mknk2, Map3k5, Map3k3, Map3k7ip2, TAB2, and ASK1. |
| 142 | DNMT1 | P26358 | 19112019 | curcumin covalently blocks the catalytic thiolate of C1226 of DNMT1 to exert its inhibitory effect. |
| 143 | MYLK | Q15746 | [21594647](http://www.ncbi.nlm.nih.gov/pubmed/21594647) | Curcumin results in increased expression of MYLK mRNA |
| 144 | BAK1 | Q16611 | [17332930](http://www.ncbi.nlm.nih.gov/pubmed/17332930) | Curcumin results in increased expression of BAK1 mRNA |
| 145 | STAT1 | P42224 | 19723881 | curcumin inhibited the phosphorylation of STAT1 |
| 146 | PTGS1 | P23219 | [20816778](http://www.ncbi.nlm.nih.gov/pubmed/20816778) | Curcumin results in decreased expression of PTGS1 mRNA |
| 147 | DRD2 | P14416 | 20513244 | Curcumin modulates dopaminergic receptor (DRD1 & DRD2) |
| 148 | IKBKE | Q14164 | 19723087 | curcumin downregulated the transcription of IKBKE |
| 149 | PTGS2 | P35354 | 17671737, 19250217 | Curcumin analog results in decreased expression of PTGS2 mRNA |
| 150 | RNF2 | Q99496 | 15470680 | The expression of some of these PcG proteins (BMI1, MEL18, PH1, RNF2, RING1, and RYBP) seems to be regulated by NFκB, and curcumin have tendency to inhibit NFκB. Which means RNF2 a type of PcG protein modulate in the presence of curcumin. |
| 151 | CTGF | P29279 | 22324466 | curcumin inhibits CTGF |
| 152 | IL6R | P08887 | 14709335, 26359498 | curcumin up-regulates disintegrin and ADAMT levels which are required for the shredding of IL6R, thus increase the shredding of IL6R |
| 153 | NGF | P01138 | 10954053 | Curcumin inhibits NGF |
| 154 | NFKB2 | Q00653 | 19723087 | curcumin downregulated the transcription of NFKB2 |
| 155 | GCLM | P48507 | 16460683 | curcumin increased the expression of GCLM |
| 156 | GSTA4 | O15217 | [10514034](http://www.ncbi.nlm.nih.gov/pubmed/10514034) | Curcumin results in increased activity of GSTA4 protein |
| 157 | JUN | P05412 | 23875250 | curcumin downregulated the expression of c-JUN |
| 158 | CYP2C19 | P33261 | 18480186 | curcuminoid extract inhibited the activity of CYP2C19 |
| 159 | VEGFA | P15692 | 22739211 | curcumin supresses the release of VEGFA |
| 160 | ABL1 | P00519 | 27608133 | curcumin shows high binding affinity towards ABL1 |
| 161 | UGT1A7 | Q9HAW7 | 15845768 | curcumin inhibits the activity of cellular UGT1A7 |
| 162 | UGT1A8 | Q9HAW9 | [14557274](http://www.ncbi.nlm.nih.gov/pubmed/14557274) | UGT1A8 protein results in increased glucuronidation of Curcumin |
| 163 | HDAC1 | Q13547 | 23430957 | curcumin inhibited HDAC1 |
| 164 | HDAC8 | Q9BY41 | 23430957 | curcumin decreased the levels of HDAC8 |
| 165 | TRAF1 | Q13077 | 19372569 | curcumin supresses the production of TRAF1 |
| 166 | TLR4 | O00206 | 29901164 | curcumin significantly increased the expression of TLR4 |
| 167 | CNR2 | P34972 | 19359525 | Curcumin selectively bind human CB1 cannabinoid receptors function as antagonists/inverse agonists |
| 168 | AR | P10275 | 12239622 | curcumin downregulates the expression of AR |
| 169 | SYK | P43405 | 20158382 | curcumin interferes with the kinase activity of SYK |
| 170 | PCNA | P12004 | [18226269](http://www.ncbi.nlm.nih.gov/pubmed/18226269) | Curcumin results in decreased expression of PCNA protein |
| 171 | EDN1 | P05305 | 30020318 | curcumin significantly downregulates EDN1 |
| 172 | FOXO1 | Q12778 | 23888319 | curucumin induced the expression of FOXO1 |
| 173 | CYP1A1 | P04798 | [20816778](http://www.ncbi.nlm.nih.gov/pubmed/20816778) | Curcumin results in decreased expression of CYP1A1 mRNA |
| 174 | RBM17 | Q96I25 | 22475723 | curcumin modulates the expression of RBM17 |
| 175 | IRF4 | Q15306 | 22110192 | curcumin inhibits IRF4 pathway |
| 176 | JAK2 | O60674 | [16959222](http://www.ncbi.nlm.nih.gov/pubmed/16959222) | Curcumin results in decreased expression of JAK2 protein |
| 177 | CDK1 | P06493 | 21617861 | curcumin decreases the expression of CDK1 |
| 178 | CAMK2B | Q13554 | 15489172 | curcumin analogs inhibit the functioning of Ca2+/CAM which further inhibits the phosphorylation of CAMK2B |
| 179 | GSTP1 | P09211 | 19350453 | curcumin inhibits GSTP1 |
| 180 | RELA | Q04206 | 12216086, 16173963, 18226269 | Curcumin results in decreased activity of RELA protein |
| 181 | TF | P02787 | 18815282 | curcumin declines the saturation level of transferrin |
| 182 | TICAM2 | Q86XR7 | 19723087 | curcumin downregulated the transcription of TICAM2 |
| 183 | CREB1 | P16220 | 15383533 | curcumin inhibits CREB1 |
| 184 | JAK3 | P52333 | 16932349 | curcumin inhibits JAK3 |
| 185 | IGF2 | P01344 | 18348204 | curcumin helps in maintaining the expression of monoallelic IGF2 |
| 186 | E2F5 | Q15329 | [15713895](http://www.ncbi.nlm.nih.gov/pubmed/15713895) | Curcumin results in decreased expression of E2F5 mRNA |
| 187 | CD44 | P16070 | 24626093 | curcumin reduced the expression of CD44 |
| 188 | TNF | P01375 | 23425071 | curcumin acts as a TNF blocker |
| 189 | MYD88 | Q99836 | 19723087 | curcumin downregulates the transcription of MYD88 |
| 190 | WEE1 | P30291 | 14534529 | curcumin decreased the amount of Wee1. |
| 191 | BRCA1 | P38398 | 23825154 | curcumin reduced the expression of BRCA1 |
| 192 | HDAC2 | Q92769 | [18421014](http://www.ncbi.nlm.nih.gov/pubmed/18421014) | Curcumin inhibits the reaction [Smoke results in decreased activity of HDAC2 protein] |
| 193 | IKBKB | O14920 | 19723087 | curcumin decreased the transcription of IKBKB |
| 194 | CDC27 | P30260 | 22280307 | curcumin targets CDC27 |
| 195 | ITGAM | P11215 | [9112257](http://www.ncbi.nlm.nih.gov/pubmed/9112257) | [Curcumin results in increased expression of ITGAM |

**Supplementary Table 2:** Molecular docking analysis of final 12 possible key regulatory proteins of curcumin rewired PIN with curcumin.

| **S. No.** | **Receptor** | **Ligand** | **Binding Energy (KCal/Mol)** | **Ki** | **Binding Residues** | **Hydrogen Bond** | **H-Bond Distance (Ǻ)** |
| --- | --- | --- | --- | --- | --- | --- | --- |
| 1 | MAPK1/Erk2 (3W55) | Curcumin | -8.43 | 662.55 nM | Ile31,Tyr36,Val39,Ala52,Ile53,Lys54  Ile84,Gln105,Asp106,Leu107,Met108  Glu109,Thr110,Asp111,Lys114,Ser153  Asn154,Leu156,Cys166,Asp167 | MAPK1:ASN154:ND2 - :Curcumin:O27 | 2.73722 |
| 2 | STAT1 (1YVL) | Curcumin | -7.68 | 2.36 µM | Trp239,Gln243,Ala246,Cys247,Asn253  Ala254,Cys255,Leu256,Glu320,Gln322  Cys324,Pro329,Gln330,Arg331,Pro332  Arg346 | STAT1:CYS247:SG - : Curcumin :O12  Curcumin :H47- STAT1:GLU320:OE2  Curcumin :H43 - STAT1:PRO329:O | 3.019  2.26351  1.98447 |
| 3 | KRAS  (5O2S) | Curcumin | -7.48 | 3.27 µM | Val12,Gly13,Lys16,Ser17,Ala18,Val29  Glu31,Tyr32,Asp33,Pro34,Asp57,Thr58  Ala59,Lys117 | KRAS:ALA18:N - : Curcumin:O12  : Curcumin:H47 - KRAS:PRO34:O | 3.0762  2.02035 |
| 4 | P53  (3KZ8) | Curcumin | -6.57 | 15.39 µM | Ser96,Val97,Pro98,Ser99,Arg158,Met160  Asp208,Thr211,Arg213,Ile254,Thr256  Glu258,Gly262,Arg267 | : Curcumin:H43 - P53:SER99:O | 2.09828 |
| 5 | CREBBP  (4TQN) | Curcumin | -6.29 | 24.67 µM | Leu1109,Pro1110,Gln1113,Val1115,Leu1120,Tyr1167,Asn1168,Arg1169,Ser1172,Arg1173,Val1174 | : Curcumin:H47 - CREBBP  :GLN1113:OE1:B | 2.4307 |
| 6 | RELA  (1NFI) | Curcumin | -6.07 | 35.49 µM | Glu211,Phe213,Leu214,Leu215,Ser238,Ala242,Asp243,His245,Ala249,Ile250,Val251,Phe252,Arg253 | N/A | N/A |
| 7 | AKT1  (4EKL) | Curcumin | -6.04 | 37.57 µM | Leu156,Gly157,Lys158,Gly159,Val164, Glu234,Phe236,Lys276,Leu277,Glu278, Met281,Thr312,Tyr315,Phe438, Asp439  Phe442 | N/A | N/A |
| 8 | CASP8 (1I4E) | Curcumin | -5.81 | 55.52 µM | Asp2223, Lys2224,Val2225,Gly2436  Asp2437,Asp2438,Leu2470,Arg2471  Lys2472,Lys2473,Val2475 | Curcumin:H43- CASP8:GLY2436:O | 1.98974 |
| 9 | CREB1  (5ZKO) | Curcumin | -5.73 | 62.94 µM | LYS292, GLU295, ALA 296, ARG 298, GLU 299, ARG301, ARG302, LYS305 | CREB1:ARG298:NE-Curcumin:O12  CREB1:ARG298:NH2-Curcumin:O12  CREB1:ARG301:NE-Curcumin:O25  CREB1:ARG302:NH1-Curcumin :O15 | 3.10094 3.18326 3.05441 3.19184 |
| 10 | NFKB1 (2O61) | Curcumin | -5.72 | 64.04 µM | His141,Leu207,Ser208,Lys241,Ala242, Pro243,Lys272,Gln274,Lys275,Gln306 | NFKB1:LYS272:HZ2 -Curcumin:O15  NFKB1:GLN274:HE21 -Curcumin:O27  Curcumin:H43 - NFKB1:LEU207:O | 1.88788  2.2961  1.92234 |
| 11 | RB1  (1AD6) | Curcumin | -5.34 | 121.27 µM | Val434,Cys438,Arg500,Gln504,Asn505,Leu506,Asp507,Ser508,Gly509,Thr510 | RB1:ASN505:HD21 -Curcumin :O2 | 2.12383 |
| 12 | SRC  (1A1C) | Curcumin | -4.85 | 279.98 µM | Arg158,Glu162,Val202,Lys203,His204,Tyr205,Lys206,Arg208,Ile217,Thr218 | : Curcumin:H43 - SRC:ILE217:O | 2.07802 |
